# Simultaneous inhibition of endocytic recycling and lysosomal fusion sensitizes cells and tissues to oligonucleotide therapeutics

**DOI:** 10.1093/nar/gkad023

**Published:** 2023-02-02

**Authors:** Brendan T Finicle, Kazumi H Eckenstein, Alexey S Revenko, Brooke A Anderson, W Brad Wan, Alison N McCracken, Daniel Gil, David A Fruman, Stephen Hanessian, Punit P Seth, Aimee L Edinger

**Affiliations:** Department of Developmental and Cell Biology, University of California Irvine, Irvine, CA, USA; Department of Developmental and Cell Biology, University of California Irvine, Irvine, CA, USA; Ionis Pharmaceuticals, Carlsbad, CA, USA; Ionis Pharmaceuticals, Carlsbad, CA, USA; Ionis Pharmaceuticals, Carlsbad, CA, USA; Siege Pharmaceuticals, Irvine, CA, USA; Siege Pharmaceuticals, Irvine, CA, USA; Department of Molecular Biology and Biochemistry, University of California Irvine, Irvine, CA, USA; Department of Chemistry, Université de Montréal, Montréal, QC, Canada; Department of Pharmaceutical Sciences, University of California Irvine, Irvine, CA, USA; Ionis Pharmaceuticals, Carlsbad, CA, USA; Department of Developmental and Cell Biology, University of California Irvine, Irvine, CA, USA

## Abstract

Inefficient endosomal escape remains the primary barrier to the broad application of oligonucleotide therapeutics. Liver uptake after systemic administration is sufficiently robust that a therapeutic effect can be achieved but targeting extrahepatic tissues remains challenging. Prior attempts to improve oligonucleotide activity using small molecules that increase the leakiness of endosomes have failed due to unacceptable toxicity. Here, we show that the well-tolerated and orally bioavailable synthetic sphingolipid analog, SH-BC-893, increases the activity of antisense oligonucleotides (ASOs) and small interfering RNAs (siRNAs) up to 200-fold in vitro without permeabilizing endosomes. SH-BC-893 treatment trapped endocytosed oligonucleotides within extra-lysosomal compartments thought to be more permeable due to frequent membrane fission and fusion events. Simultaneous disruption of ARF6-dependent endocytic recycling and PIKfyve-dependent lysosomal fusion was necessary and sufficient for SH-BC-893 to increase non-lysosomal oligonucleotide levels and enhance their activity. In mice, oral administration of SH-BC-893 increased ASO potency in the liver by 15-fold without toxicity. More importantly, SH-BC-893 enabled target RNA knockdown in the CNS and lungs of mice treated subcutaneously with cholesterol-functionalized duplexed oligonucleotides or unmodified ASOs, respectively. Together, these results establish the feasibility of using a small molecule that disrupts endolysosomal trafficking to improve the activity of oligonucleotides in extrahepatic tissues.

## Graphical Abstract

**Graphical Abstract:**
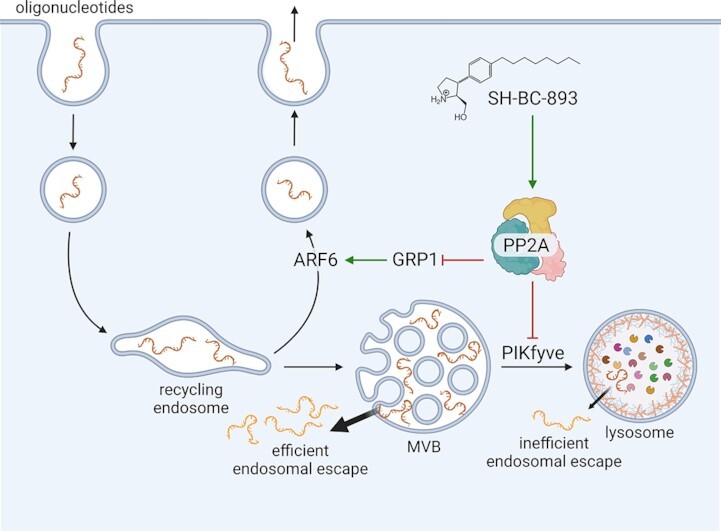
We show that the small molecule SH-BC-893 increases intracellular oligonucleotide levels boosting activity >100-fold *in vitro* and >10-fold *in vivo* by simultaneously limiting endosomal recycling and lysosomal accumulation of oligonucleotides.

## INTRODUCTION

Oligonucleotide therapeutics have the potential to revolutionize medicine by making almost any target accessible. Oligonucleotides targeting RNA include single-stranded antisense oligonucleotides (ASOs) that base pair with a target RNA to elicit RNaseH-dependent degradation, inhibition of translation, or changes to splicing (1,2). Double-stranded small interfering RNAs (siRNAs) degrade target RNAs after being loaded into the RNA-induced silencing complex (RISC). Medicinal chemistry optimization of the drug-like properties of ASOs and siRNAs solved historical problems with stability and rapid clearance. At least 13 therapeutic oligonucleotides have been FDA-approved and hundreds are in preclinical development (3–8).

Despite these successes, inefficient delivery to targets in the cytosol and nucleus of cells remains a major barrier to the broad application of oligonucleotide therapeutics (2,9–11). Only 1% of the oligonucleotide that is delivered to patients engages its target. The high concentrations required in target tissues are readily achieved in the liver after subcutaneous administration. Extrahepatic tissues generally require local (e.g. intrathecal or aerosol) delivery or frequent administration of high doses to achieve significant target engagement (9). Conjugation to ligands that bind to cell surface receptors can increase oligonucleotide activity by increasing cellular uptake (9,12,13). Thus far, the only liganded oligonucleotides that are FDA-approved are *N*-acetylgalactosamine (GalNAc) conjugates that target hepatocytes. Other ligand-target pairs will require optimization for extrahepatic delivery. Approaches that address post-endocytic blocks to delivery offer an alternate strategy to extend the range of tissues and cells that are accessible to oligonucleotide therapeutics.

As large (4–14 kDa) polar molecules, ASOs and siRNAs do not readily diffuse across lipid bilayers; both receptor-targeted and unconjugated oligonucleotides enter cells via endocytosis (9,10). Oligonucleotides in endocytic vesicles are either recycled back to the extracellular space through exocytosis or progress to lysosomes, the degradative compartment of the cell (9,14,15). Chemical modifications render therapeutic oligonucleotides resistant to lysosomal nucleases (2,9,10). Therefore, the majority of endocytosed oligonucleotides accumulate within lysosomes where they are stable but unable to reach their cytosolic targets, although recent evidence suggests slow leakage from lysosomes supports long-term oligonucleotide activity in the liver (16). Attempts to improve the escape of oligonucleotides from endosomes and lysosomes into the cytosol have met little success. Genetic knockdown screens have failed to identify targetable proteins that significantly enhance oligonucleotide activity (17,18). Molecules that increase the leakiness of endosomes produce large increases in oligonucleotide activity but have a narrow therapeutic index because permeabilizing endosomes and lysosomes is toxic (19–25). Therefore, novel approaches that improve oligonucleotide escape into the cytosol without lysing endocytic compartments are required to solve the delivery problem that limits the therapeutic use of oligonucleotides.

In the absence of permeabilizing agents, oligonucleotides likely escape from endocytic compartments at sites of membrane fission and fusion (18,26–28). During these dynamic membrane remodeling events, the lipid bilayer is deformed to create non-bilayer regions that have increased permeability (29–33). Consistent with this model, pre-lysosomal compartments that undergo high rates of vesicle budding and fusion have been identified as sites of oligonucleotide escape (17,34–36). Escape from lysosomes is much less efficient because the limiting membrane is heavily decorated with glycoproteins (e.g. LAMP1 and LAMP2) and glycolipids that reduce permeability relative to other endocytic structures (37,38). Approaches that increase oligonucleotide uptake and/or residency time in pre-lysosomal compartments where oligonucleotide escape is most efficient could offer significant gains in potency that would make extrahepatic tissues therapeutically accessible.

**Figure 1.**
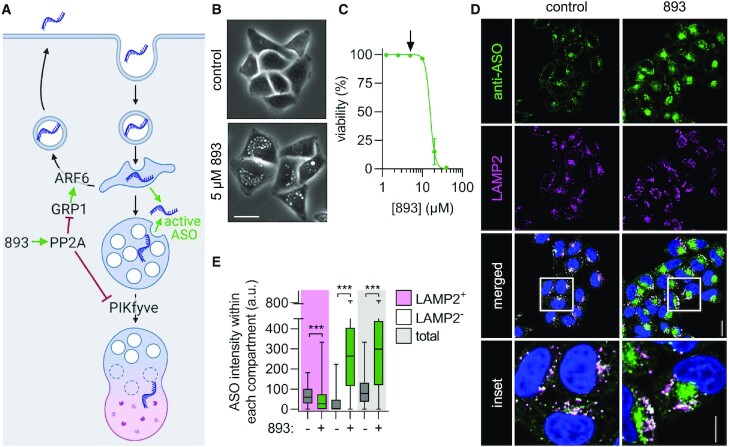
SH-BC-893 increases oligonucleotide accumulation in non-lysosomal compartments. (**A**) Model showing how SH-BC-893 (893) alters intracellular trafficking. (**B**) Phase contrast images of HeLa cells treated with SH-BC-893 (5 μM) for 3 h. Scale bar = 20 μm. (**C**) Viability measured by vital dye (DAPI) exclusion through flow cytometry in HeLa cells treated with indicated concentrations of SH-BC-893 for 24 h. Arrow indicates concentration used in all in vitro oligonucleotide assays. Mean ± SD shown, *n* = 3. (**D**) HeLa cells treated with a 3–10–3 cEt ASO targeting *MALAT1* (2 μM) ± SH-BC-893 (5 μM) for 6 h and stained with antibodies to endogenous LAMP2 or PS-ASOs. Scale bar = 20 μm. For inset, scale bar = 10 μm. (**E**) Quantification of the raw intensity values for ASO from images in (D) within LAMP2-positive, LAMP2-negative, and total cellular areas. At least 100 cells were quantified from each of 2 independent experiments. Using a Mann–Whitney *t* test to correct for data that is not normally distributed, ***P < 0.001.

We have published that the synthetic sphingosine analog SH-BC-893 disrupts endocytic recycling by inactivating the small GTPase ARF6 and blocks lysosomal fusion reactions that depend on the lipid kinase PIKfyve (39–42). We hypothesized that simultaneous disruption of these endolysosomal trafficking pathways would synergistically increase oligonucleotide activity by causing accumulation within pre-lysosomal compartments where endosomal release is most efficient (Figure 1A). Importantly, these changes in trafficking are well tolerated as SH-BC-893 is non-toxic at the effective dose even with chronic administration (39–41). Here, we demonstrate that the parallel actions of SH-BC-893 on endocytic recycling and lysosomal fusion are necessary and sufficient to increase intracellular oligonucleotide levels in extra-lysosomal compartments and significantly enhance oligonucleotide activity both in vitro and in vivo with no toxicities detected.

## MATERIALS AND METHODS

### Cell lines and cell culture

MDA-MB-468, MDA-MB-231, SW620, NCI-H358, A549, BxPC3 and PANC1 were obtained from the ATCC. HeLa cells were obtained from Steve Caplan (University of Nebraska Medical Center, Omaha, NE, USA). *p53*^–/–^ MEFs were generated in-house from embryos from C57BL/6 mice (stock no. 008462, The Jackson Laboratory) using standard techniques. All cells were maintained at 37°C in 5% CO_2_. HeLa, MEFs, A549, BxPC3 and PANC1 cells were cultured in DMEM media supplemented with 10% fetal bovine serum (FBS) without antibiotics. MDA-MB-468, MDA-MB-231 and SW620 were cultured in DMEM media supplemented with 10% FBS and 1% sodium pyruvate without antibiotics. All cells were maintained in culture for no more than 3 weeks before low-passage vials were thawed. *Mycoplasma* testing was performed monthly using the published PCR protocol from Uphoff and Drexeler (43).

### Chemicals and reagents

SH-BC-893 was synthesized by IntelliSyn RD (Montreal, Quebec, Canada) following the methods in (44) and with advice from S. Hanessian. The following chemicals were purchased: YM201636 (Cayman Chemicals, cat# 13576), apilimod (SelleckChem, cat# S6414), NAV2729 (R&D systems, cat# 5986), SecinH3 (Cayman Chemicals, cat# 10009570), perphenazine (PPZ, Sigma, cat# P6402-1G), UNC10217938A (Medchemexpress, cat# HY-136151), 6BIO (Cayman Chemicals, cat# 13123), AZD8055 (Cayman Chemicals, cat# 16978) and retro-2 (Sigma, cat# SML1085-5MG). Stock solutions were prepared as follows, aliquoted, and stored at –20°C: SH-BC-893 (5 mM in H_2_O), YM201636 (1.6 mM in DMSO), apilimod (100 μM in DMSO), NAV2729 (12.5 mM in DMSO), SecinH3 (30 mM in DMSO), perphenazine (50 mM in DMSO), UNC10217938A (10 mM in DMSO), 6BIO (15 mM in DMSO), AZD8055 (1 mM in DMSO), and retro-2 (100 mM in DMSO). All chemical structures of compounds are shown in Supplementary Figure S1. Oligonucleotides used are shown in Supplementary Tables 1, 3 and 4 and were obtained from Ionis Pharmaceuticals.

### Fluorescence microscopy sample preparation

Cells were seeded into glass bottom 8-chamber slides at 12 000 cells per chamber (Cellvis, cat# C8-1.5H-N). 16–24 h after seeding, cells were incubated with ASOs (2 μM) for indicated time points. Post-incubation, cells were washed three times with PBS, fixed in 4% paraformaldehyde (VWR, cat# 100503–917) for 15 min, and then washed again with PBS. For samples where no other proteins were immunostained, cells were imaged following DAPI staining (1 mg/ml in PBS, VWR, cat# 422801-BL) for 5 min. For samples where other proteins or molecules were immunostained (e.g. LAMP1, LAMP2, PS-ASOs or myc-tag), samples were permeabilized and incubated in blocking solution for 30 min at RT (10% FCS, 0.3% saponin, 0.05% azide in PBS). Samples were incubated with primary antibody diluted in block solution for 1 h at RT or overnight at 4°C, washed three times with PBS, and then stained in Alexa Fluor-conjugated secondary antibody solution at RT for 1 h with rocking. Samples were washed again three times, stained with DAPI, washed, and then imaged in PBS by confocal microscopy. Antibodies: anti-LAMP1 (Cell Signaling Technologies, cat# 9091S, 1:400 dilution), anti-LAMP2 (Developmental Studies Hybridoma Bank, cat# H4B4, 1:200 dilution), anti-PS-ASOs (provided by Ionis Pharmaceuticals, 1:200 dilution), anti-myc-tag (Cell Signaling Technologies, cat# 2278S, 1:200 dilution), Alexa Fluor 594 goat anti-mouse (Fisher Scientific, cat# A11032, 1:200 dilution), and Alexa Fluor 594 goat anti-rabbit (Fisher Scientific, cat# A-11012, 1:200 dilution).

### Microscopy, image analysis, and quantification

All microscopy images were collected with ZEN digital imaging software on a Zeiss LSM780 confocal microscope with a Plan-Apochromat 63×/1.40 Oil DIC objective or a Zeiss LSM900 with Airyscan 2 with a Plan-Apochromat 63×/1.40 Oil DIC objective. All quantification of microscopy data was performed using ImageJ. In brief, regions of interest (ROIs) enclosing individual cells in each field of view were drawn using cell autofluorescence to define cell boundaries. LAMP1/2-positive area was defined by turning LAMP1 or LAMP2 fluorescent images into a binary image and utilizing ImageJ to automatically draw a ROI around each LAMP1- or LAMP2-positive lysosome. LAMP1/2-negative area was defined by subtracting the LAMP1/2-positive ROI from the ROI enclosing the total cell. ASO fluorescence that did or did not colocalize with LAMP1/2-positive pixels was measured as Integrated Density per cell. Total intracellular ASO fluorescence was measured by adding the fluorescence of ASOs within LAMP1/2-positive or LAMP1/2-negative ROIs. For cytoplasmic ASO quantification, endosomal ASO signal was eliminated by generating regions of interest (ROIs) on thresholded images. With the diffuse cytoplasmic signal remaining, the Integrated Density per cell was measured. Background subtraction was performed by quantifying fluorescent signal in cells that were not exposed to ASOs but stained with both primary and secondary antibodies. At least 100 cells per experiment from a total of 2–3 independent experiments were analyzed for each experiment.

### RNA isolation and RT-qPCR

To monitor ASO or siRNA activity *in vitro*, 3000 cells were plated in duplicate or triplicate wells of a 96-well flat bottom plate. After 16–24 h, cells were treated with a serial dilution of ASOs starting at 20 μM and including 3-fold serial dilutions. Cells were lysed 24 h after ASO addition in GTC lysis buffer (4 M guanidine isothiocyanate, 50 mM Tris–HCl pH 7.5, 25 mM EDTA). RNA was immediately purified or lysates were stored in this buffer at –20°C for later purification. Samples were mixed with an equal amount of 70% EtOH and pipetted into a Pall 96-well glass fiber filter plate (VWR, cat# 97052-104) fitted onto a vacuum manifold. Samples were then washed twice with RNA wash buffer (60 mM potassium acetate, 10 mM Tris–HCl pH 7.5, 60% EtOH) before digesting genomic DNA using DNaseI (Fisher, cat# 18047019) in DNaseI buffer (400 mM Tris–HCl pH 7.5, 100 mM NaCl, 100 mM CaCl_2_, 100 mM MgCl_2_) for 15 min at RT. Samples were then washed twice with GTC wash buffer (1 M guanidine isothiocyanate, 12.5 mM Tris–HCl (pH 7.5), 6.25 mM EDTA) followed by three washes in RNA wash buffer. After the final wash, residual buffers were removed from the glass fiber plate by spinning in a tabletop centrifuge for 5 min at 3696 × g. RNA was eluted in nuclease-free water into a 96-well round bottom plate. Following RNA elution, RT-qPCR reaction plates were set up by loading 5 μl of RNA into each well and using the Taqman AgPath-ID™ One-Step RT-PCR Reagents (Fisher, cat# 4387391) according to supplier's recommendations. Taqman primers and probes are described in Supplementary Table 2. RNA levels were quantified using a StepOnePlus Real-Time PCR system (Applied Biosystems) and normalized to total RNA loaded using Quant-iT™ RiboGreen™ RNA Assay Kit (Invitrogen, cat# R11490) according to supplier's recommendations. RNA levels are expressed relative to control or drug-treated samples that received no ASOs. Two to three technical replicates each were performed for each ASO activity assay. At least three independent experiments were performed to produce biological replicates.

To monitor ASO activity in tissues in vivo, ∼10–100 mg of flash-frozen tissue were lysed in 1 ml of Trizol using a Bullet Blender Storm Pro Tissue Homogenizer (Next Advance, cat# BT24M). After complete tissue homogenization, samples were transferred to a new Eppendorf tube and incubated at RT for 5 min to permit complete dissociation of nucleoprotein complexes. Following addition of 0.2 ml of chloroform to the Trizol-homogenate, the tube was inverted vigorously 3–4 times and then by vortexing for 20 s. Samples were then centrifuged at 13 000 rpm in a microcentrifuge at 4°C for at least 15 min. Following centrifugation, the upper aqueous layer containing RNA was transferred to a new tube. RNA was precipitated by adding 0.5 ml of ice-cold isopropanol followed by centrifugation at 13 000 rpm in a microcentrifuge at 4°C for 30–60 min. The resulting RNA pellet was washed twice with 1 ml of 70% EtOH (centrifuging for 5 min at 13 000 rpm to clear washes). The washed RNA pellet was dissolved in 40–100 μl of nuclease-free water, and then quantified using a NanoDrop™ 2000 spectrophotometer. To achieve pure RNA, approximately 10 μg of RNA was treated with DNaseI (Zymo Research Corporation, cat# E1010) and then cleaned up on spin columns (New England Biolabs, cat# T2030L) according to the supplier's recommendations. qRT-PCR reaction plates were set up by loading 50 ng of RNA into each well and using the Taqman AgPath-ID™ One-Step RT-PCR Reagents (Fisher, cat# 4387391) according to the supplier's recommendations. Taqman primers and probes are described in Supplementary Table 2. RNA levels were quantified using a StepOnePlus Real-Time PCR system (Applied Biosystems) and expressed relative to housekeeping gene *Ppia* using the 2^−ΔΔCt^ method prior to normalization as described in the figure legends.

### Viability measurement by flow cytometry

Viability was measured by vital dye exclusion using DAPI. In brief, adherent cells were plated in 24-well plates at 30 000 cells per well (matching the confluency of the cells in 96-well plates in the RT-qPCR experiments). After 16–24 h, cells were treated with indicated compounds at indicated concentrations for 24 h. After 24 h, the following was collected in FACS tubes: media containing floating dead cells, a PBS wash, trypsinized cells, and a final media wash to collect all remaining cells and debris. This live and dead cell containing solution was pelleted by centrifugation and the supernatant discarded. The pellet was placed back in single-cell suspension with 0.6 ml of media containing 1 μg/ml of DAPI. Samples were evaluated on a BD Fortessa X20.

### Plasmids and stable cell line generation

pQCXIP-mCherry and pQCXIP-mCherry-VAC14 were generously provided by Thomas Weide (University Hospital of Muenster, Germany). The pQCXIB Firefly Luciferase plasmid was a gift from Reuben Shaw (Addgene plasmid # 74445; http://n2t.net/addgene:74445; RRID:Addgene_74445). myc-GRP1^DD^ was produced by PCR amplification of the insert in pEF6-myc-GRP^S155D/T240D^ (a generous gift from Victor Hsu, Harvard Medical School, Boston, MA, USA) and subsequent Gateway cloning into pQCXIB CMV/TO DEST (39–41), a gift from Eric Campeau & Paul Kaufman (Addgene plasmid # 17400; http://n2t.net/addgene:17400; RRID:Addgene_17400). Stable cell lines were generated by transducing target cells with retrovirus and drug selection.

### General animal procedures

All experiments measuring ASO activity in animals were approved by the Institutional Animal Care and Use Committee of University of California, Irvine. Six- to eight-week old, male Balbc/J mice were purchased from the Jackson Laboratory and acclimated to the university vivarium for at least 7 days prior to experimentation. Mice were housed under a 12 h light/dark cycle at 20–22°C in groups of 2–4. Cages contained 1/8′ corncob bedding (7092A, Envigo, Huntingdon, UK) enriched with ∼6 g of cotton fiber nestlets (Ancare, Corp., Bellmore, NY). Mice were fed the vivarium stock diet (chow, 2020x, Envigo). Access to food and water was ad libitum. For oral administration of SH-BC-893, polypropylene feeding tubes (20 g × 38 mm; Instech Laboratories Inc., Plymouth, PA) were used to dose 120 mg/kg of SH-BC-893 dissolved in H_2_O (stock = 24 mg/ml). To aid gavage by inducing salivation, feeding tubes were dipped into a 1 g/ml sucrose solution immediately prior to treatment. For subcutaneous administration, ASOs were dissolved in PBS at a concentration such that 10 ml/kg was administered using a 27 G needle. For blood chemistry analysis, blood was collected by decapitation from nine-week old male Balbc/J mice. Serum was separated from whole blood in a SST-MINI tube with clot activator gel (Greiner, cat# 450571VET). Serum samples were sent to IDEXX Bioanalytics for a comprehensive chemistry panel (cat# 6006).

### Tissue distribution and pharmacokinetics of SH-BC-893

Tissue PK studies were performed at Pharmaron (Beijing, China) on a fee-for-service basis and were approved by their Institutional Animal Care and Use Committee. Six- to eight-week old male or female CD1 mice were treated daily for 5 days with 120 mg/kg SH-BC-893 dissolved in H_2_O (P.O., stock = 12 mg/ml) and then sacrificed 0.5, 1, 2, 4, 8 or 24 h after the last dose. Tissues were perfused with 10 mL saline and prior to collection and snap frozen in liquid nitrogen. Frozen tissue was homogenized in PBS (W/V 1:4). 10 μl of tissue or plasma homogenate was mixed with 10 μl blank solution and added to 200 μl acetonitrile containing FTY720 as an internal standard. Samples were vortexed and then spun for 30 min at 4700 rpm at 4°C to precipitate protein. The resultant supernatant was diluted 2-fold with water and 10 μl of diluted supernatant was injected into the LC–MS/MS for quantitative analysis. SH-BC-893 was quantified by LC–MS/MS using a HALO 90A AQ-C18, 2.7 μm 2.1 × 50 mm column and an AB Sciex Triple Quad 5500 LC–MS/MS instrument (serial no. BB214861610) and corrected for extraction efficiency using the FTY720 internal standard.

### Statistical analysis

For microscopy experiments, box and whisker plots showing median and quartiles are presented because data was not normally distributed. In bar graphs depicting ASO or siRNA IC50s or target RNA levels in tissues, mean ± SD is presented. All experimental data are from ≥ 3 independent biological replicates except where otherwise indicated. Statistical analysis was performed using GraphPad Prism software. Corrections for multiple comparisons were made as indicated in the legends and adjusted P-values reported: n.s., not significant, *P* ≥ 0.05; * *P* < 0.05; ** *P* < 0.01; *** *P* < 0.001; key comparisons are shown in the figures.

## RESULTS

### SH-BC-893 increases ASO delivery and activity *in vitro*

SH-BC-893 (Supplementary Figure S1) disrupts endocytic recycling by inactivating the small GTPase ARF6 and blocks lysosomal fusion reactions that depend on the lipid kinase PIKfyve (39–42), activities that we predicted would synergize to promote oligonucleotide delivery (Figure 1A). HeLa cervical carcinoma cells were used initially to test this hypothesis because they are well suited to confocal microscopy and widely used to study both intracellular trafficking and oligonucleotide delivery. As expected, SH-BC-893 disrupted endolysosomal trafficking in HeLa cells at non-toxic concentrations (Figure 1B, C). While toxic in vitro at high concentrations, SH-BC-893 is well tolerated in mice at the effective dose (120 mg/kg) even with chronic administration (39,41). Initial studies used a phosphorothioate (PS) gapmer complementary to the long non-coding RNA (lncRNA) *MALAT1* with 10 deoxynucleotides in the center flanked on each side by 3 nucleotides containing riboses with a 2′,4′ constrained ethyl (cEt) group (Supplementary Table S1); this modification is common among preclinical ASOs and is currently being evaluated in patients (9). ASO localization was tracked using a polyclonal antibody specific for PS ASOs (17).

ASO localization was evaluated by confocal fluorescence microscopy after gymnotic delivery (‘free uptake’). In control cells, the majority of internalized ASOs colocalized with the lysosomal marker LAMP2 after 6 h (Figure 1D, E and Supplementary Figure S2A). Co-treatment with SH-BC-893 (5 μM) reduced the percent of ASOs in LAMP2-positive lysosomes from 70% to 9%. Moreover, SH-BC-893 treatment increased the total amount of intracellular ASOs by 4-fold (Figure 1D, E). Reducing the delivery of ASOs to LAMP2-positive lysosomes and concomitantly increasing intracellular ASO levels translated to a >250-fold increase in the amount of ASOs in extra-lysosomal, LAMP2-negative compartments. Similar results were obtained with an untagged 2′-*O*-methoxyethyl (2′MOE) PS-gapmer, an ASO tagged on the 5’ end with a 6-carboxyfluorescein (FAM) fluorophore, and an antibody against another lysosomal marker, LAMP1 (Supplementary Table 1 and Supplementary Figure S2B–G). In sum, co-incubation with SH-BC-893 increased the total amount of intracellular ASOs and dramatically reduced colocalization with lysosomal markers.

ASOs must escape from endosomal structures to produce knockdown. It is estimated that 1–4% of the oligonucleotides that are endocytosed escape to the cytoplasm (10,45,46). Such a small quantity is hard to observe in fluorescence microscopy images that contain very bright endosomal signals. To better illustrate changes in the low, cytoplasmic levels of ASOs, the endosomal ASO signal was removed and the cytoplasmic signal quantified (Supplementary Data Figure S3A). Background subtraction was performed using cells that were stained with both primary and secondary antibodies but not exposed to ASOs. In control cells, ∼3% of the ASOs were cytoplasmic which is consistent with prior estimates (Supplementary Figure S3B, C and (10,45,46)). SH-BC-893 treatment increased the cytosolic fraction to 10% which, given the increase in intracellular ASOs, translated to a 9-fold increase in the absolute amount of cytoplasmic ASO (Supplementary Figure S3B–D). In summary, SH-BC-893 significantly increased the amount of ASO that reaches the cytoplasm.

**Figure 2.**
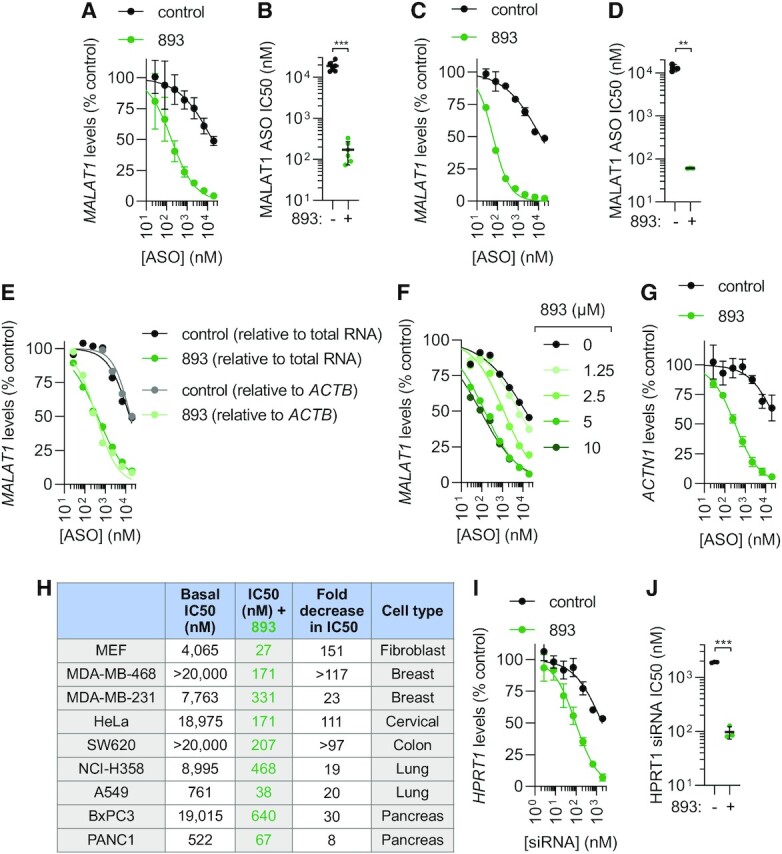
SH-BC-893 enhances oligonucleotide activity. (**A**) *MALAT1* levels in HeLa cells treated with the indicated concentrations of 3–10–3 cEt gapmer targeting *MALAT1* ± SH-BC-893 (5 μM) for 24 h. Mean ± SD shown, *n* = 6. (**B**) IC_50_s from each biological replicate in (A); mean ± SD shown. Using a Welch's *t* test to correct for unequal SD, ***P < 0.001. (**C**, **D**) As in (A, B), except using a 5–10–5 2’MOE gapmer; mean ± SD shown, *n* = 3. Using a Welch's *t* test to correct for unequal SD’s, **P < 0.01. (**E**) As in (A), except data expressed relative to total RNA or to housekeeping gene *ACTB*, *n* = 1. (**F**) As in (A), except with indicated concentrations of 893, *n* = 1. (**G**) As in (A), except using a 3–10–3 cEt gapmer targeting the *ACTN1* mRNA and *n* = 3. IC50 values for control could not be calculated due to the low basal activity of this ASO. (**H**) The indicated cell lines were treated with the cEt *MALAT1* ASO ± SH-BC-893 (5 μM) for 24 h and an IC_50_ (nM) calculated. Dose response curves shown in Supplementary Figure S5. (**I**) *HPRT1* mRNA levels in HeLa cells treated with the indicated doses of a palmitate-conjugated siRNA targeting *HPRT1* ± SH-BC-893 (5 μM) for 24 h. Mean ± SD shown, *n* = 3. (**J**) IC50s from each biological replicate in (I); mean ± SD shown. Using a Welch's *t* test to correct for unequal SD, ***P < 0.001.

Whether this increase in cytoplasmic ASOs translated into increased target RNA degradation was assessed by RT-qPCR under the same experimental conditions. Co-treatment with SH-BC-893 increased *MALAT1* knockdown in cells treated with a cEt gapmer ASO (2 μM) from 25% to 85% (Supplementary Figure S4A); similar potentiation was observed with a 2’MOE gapmer ASO (Supplementary Figure S4B). To quantify the degree of potentiation more rigorously (47), IC50s were determined for both cEt and 2’MOE ASOs in the presence or absence of SH-BC-893. With gymnotic delivery in HeLa cells, cEt and 2’MOE gapmer IC50s were 19 μM and 13 μM, respectively (Figure 2A–D). Co-incubation with SH-BC-893 shifted the dose response curves to the left by 2 logs, reducing the ASO IC50s to 171 nM (cEt) or 61 nM (2’MOE), a 111- or 215-fold increase in activity. ASO potentiation by SH-BC-893 was equally robust when normalized to total RNA or to the housekeeping gene *ACTB* (Figure 2E). Additionally, ASO potentiation by SH-BC-893 was dose responsive, ranging from an 11-fold increase in ASO activity at 2.5 μM to a ∼100-fold increase at 5 and 10 μM (Figure 2F). Notably, SH-BC-893-mediated potentiation plateaued at 5 μM, a non-toxic concentration (Figure 1C).

In our experience, the MALAT1 ASOs widely used in proof-of-concept studies are 2–3-fold more potent than most ASOs targeting other sequences. Importantly, SH-BC-893 increased the activity of oligonucleotides of other sequences in different cell types. SH-BC-893 enhanced the activity of a cEt gapmer targeting α-actinin-1 (ACTN1) >63-fold (Figure 2G). In addition to HeLa cells, potentiation was observed in SH-BC-893-treated mouse embryonic fibroblasts and in cancer cell lines derived from tumors of the breast, colon, lung, or pancreas (Figure 2H and Supplementary Figure S5). The same barriers limit ASO and siRNA delivery to their targets in the cytosol and nucleus (14,48). SH-BC-893 also improved the activity of a palmitate-conjugated, nuclease-resistant siRNA targeting *HPRT1* by 20-fold, shifting the IC_50_ from 1.9 μM to 97 nM (Figure 2I, J). These results suggest that SH-BC-893 could improve oligonucleotide activity across multiple platforms and in extrahepatic tissues.

### SH-BC-893 is less toxic or more effective than previously identified oligonucleotide-potentiating small molecules

Small molecules that enhance endosomal release of oligonucleotides through lysis have been described. For example, the small molecule UNC10217938A dramatically enhances the activity of ASOs, siRNAs, and splice-switching oligonucleotides by permeabilizing endosomes and lysosomes (19). However, the therapeutic use of endolytic agents is limited by their toxicity. Under our standard assay conditions where cells are continuously exposed to ASOs and compound for 24 h, the effective dose of UNC10217938A (19) was cytotoxic, killing 90% of the cells (Supplementary Figure S6A, B). In (19), toxicity was avoided by pre-loading cells with ASOs for 16 h and then pulsing cells with UNC10217938A for only 2 h. Under these conditions, UNC10217938A produced profound ASO potentiation without cell death (Supplementary Figure S6C, D). In contrast, SH-BC-893 did not increase ASO activity when added after ASO had reached lysosomes. SH-BC-893’s lack of toxicity at the efficacious dose (Figure 1C, Figure 2F, and Supplementary Figure S6B) and the observation that SH-BC-893 is not effective once ASO are sequestered in lysosomes (Supplementary Fig. S6D) suggests that SH-BC-893 does not function as an endolytic agent like UNC10217938A. To directly test whether SH-BC-893 permeabilizes endosomal membranes, cells pre-loaded with ASO and 10 kDa dextran were evaluated by microscopy for release into the cytosol and nucleus (Supplementary Figure S6E,F). At their effective doses, UNC10217938A, but not SH-BC-893, released both ASOs and dextran from endosomes. Together, this data shows that SH-BC-893 does not enhance ASO activity by permeabilizing lysosomes and is consistent with our proposed mechanism of action: trapping ASOs in a pre-lysosomal compartment from which oligonucleotide escape is naturally more efficient (Figure 1A).

Several other small molecules have been reported to enhance ASO and/or siRNA activity without permeabilizing endosomes or lysosomes. For example, the GSK3 inhibitor 6BIO enhances ASO and siRNA activity through an unknown mechanism (23), the mTOR kinase inhibitor AZD8055 increases ASO activity by stimulating autophagy (49), and the retrograde trafficking inhibitor retro-1 increases the activity of ASOs (21). Because the assay conditions and cell lines used in these publications vary, we directly compared the ASO potentiating ability of these compounds and SH-BC-893 in HeLa cells treated with an RNaseH-dependent ASO. Notably, many prior studies utilized splice-switching ASOs that trigger expression of luciferase or GFP, reporter assays that give a larger fold-change in activity than would an equivalent effect size in an RNaseH-dependent assay measuring RNA knockdown. Using the concentrations employed in the prior publications, 6BIO, AZD8055 and retro-2 enhanced ASO activity 3-, 4- or 5-fold, respectively, while SH-BC-893 increased ASO activity 130-fold under the same conditions (Supplementary Figure S6G-H); the structurally-related molecule retro-2 was utilized because retro-1 is not commercially available (50). None of these molecules were cytotoxic under these assay conditions (Supplementary Figure S6I). In summary, SH-BC-893 is less toxic than endosome-permeabilizing agents and more effective than previously identified small molecule ASO potentiators that do not lyse endocytic structures.

### Simultaneous PIKfyve and ARF6 inhibition is both necessary and sufficient for ASO potentiation by SH-BC-893

The mechanism by which SH-BC-893 promotes antisense activity was next evaluated. We hypothesized that the previously reported effects of SH-BC-893 on endocytic trafficking, disrupting ARF6-dependent endocytic recycling and PIKfyve-dependent lysosomal fusion reactions (39,40), were responsible for the observed oligonucleotide potentiation (Figure 1A). To test this model, the impact of selective PIKfyve (YM201636 and apilimod) or ARF6 (SecinH3 and NAV2729) inhibitors on ASO uptake and localization was compared to SH-BC-893 (structures provided in Supplementary Figure S1). These structurally distinct inhibitors are unlikely to have the same off-target effects as each other or as SH-BC-893, thereby increasing confidence that any shared effects on oligonucleotide trafficking and activity are due to ARF6 or PIKfyve inhibition.

**Figure 3.**
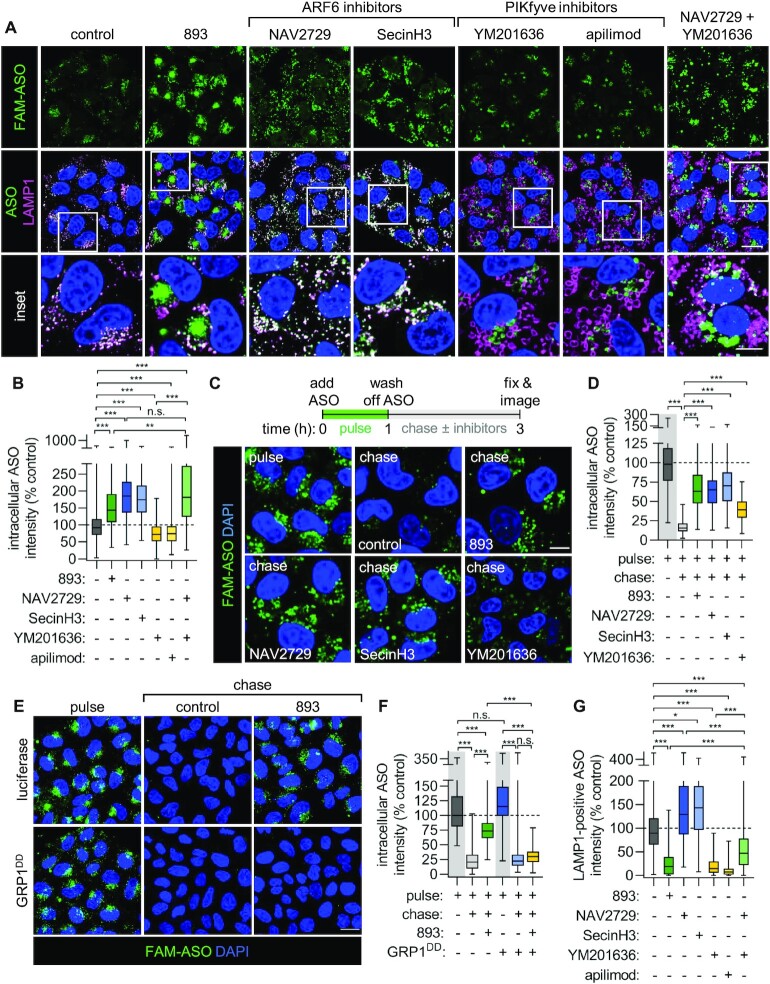
Simultaneous PIKfyve and ARF6 inhibition is both necessary and sufficient to recapitulate the effects of SH-BC-893 on ASO uptake and localization. (**A**) FAM-tagged cEt 3–10–3 ASO and LAMP1 localization in HeLa cells treated with SH-BC-893 (5 μM), NAV2729 (12.5 μM), SecinH3 (30 μM), YM201636 (800 nM), apilimod (100 nM), or both NAV2729 and YM20636 for 6 h. (**B**) Quantification of the total intracellular ASO fluorescence intensity from the images in (A). At least 100 cells were quantified from each of 3–4 independent experiments. Because the data is not normally distributed, a Kruskal–Wallis ANOVA was used with Dunn's test to correct for multiple comparisons. ***P < 0.001. (**C**) HeLa cells were pulsed with FAM-tagged 3–10–3 cEt ASO (2 μM) for 1 h, washed, and then chased in media containing vehicle (DMSO), SH-BC-893 (5 μM), NAV2729 (12.5 μM), SecinH3 (30 μM), or YM201636 (800 nM) for 2 h prior to imaging. (**D**) Quantification of the intracellular ASO fluorescence of cells in (C). At least 100 cells were quantified from each of two independent experiments. Because data is not normally distributed, a Kruskal–Wallis ANOVA was used with Dunn's test to correct for multiple comparisons. ***P < 0.001. (**E**) HeLa cells expressing luciferase or GRP1^DD^ were subjected to an ASO pulse-chase as in (C). (**F**) Quantification of the intracellular ASO fluorescence intensity in (E) performed as in (D). Scale bars, 20 μm (A and E) or 10 μm (inset in A and in C). (**G**) As in (B), except ASO fluorescence intensity within LAMP1-positive pixels is measured. **P < 0.01; ***P < 0.001.

At concentrations previously established to fully inhibit their targets (39,40), the two ARF6 inhibitors increased intracellular ASO levels to a similar extent as SH-BC-893 (Figure 3A, B). In contrast, PIKfyve inhibition with YM201636 or apilimod slightly decreased ASO accumulation within cells. ARF6 promotes endosomal recycling (15,40). To determine whether ARF6 inhibition increased intracellular ASO levels by reducing their recycling out of the cell (Figure 1A), a pulse-chase protocol was designed. Cells were pulsed with ASOs for 1 h in the absence of inhibitors, washed, and then maintained in medium lacking ASOs but containing vehicle, SH-BC-893, an ARF6 inhibitor, or a PIKfyve inhibitor for an additional 2 h (Figure 3C, D). Control cells treated with vehicle lost >80% of the ASO signal during the 2 h chase indicating that much of the ASO that enters cells is recycled. The loss of signal during the chase was not due to quenching of the 6-FAM fluorophore as similar results were obtained with untagged ASOs detected with an antibody recognizing PS ASOs (Supplementary Figure S7A, B). In contrast to the >80% loss of signal in control cells, only 25–30% of the ASO signal was lost when cells were chased in medium containing SH-BC-893 or the ARF6 inhibitors SecinH3 and NAV2729 (Figure 3C, D). Thus, ARF6 inhibition is sufficient to account for SH-BC-893’s ability to increase intracellular ASO levels and reduce ASO recycling.

Results using ARF6 inhibitors were validated with genetic approaches. The classic knockdown/knockout target validation experiments could not be performed because knockdown of ARF6 compromised cellular health to an extent that would confound the interpretation of results. We have previously shown that inhibition of ARF6-mediated endocytic recycling by SH-BC-893 occurs downstream of activation of the serine and threonine protein phosphatase 2A (PP2A) (40). ARF6 is activated by its guanine nucleotide exchange factor GRP1 which is in turn inactivated by PP2A-dependent dephosphorylation (40,51). Replacing serine 255 and threonine 280 with phosphomimetic aspartic acid residues (GRP1^DD^) renders GRP1 resistant to inactivation by PP2A and restores recycling in SH-BC-893-treated cells. Consistent with a model where SH-BC-893 increases intracellular ASO levels by inactivating ARF6, SH-BC-893 failed to block ASO recycling in GRP1^DD^ expressing cells (Figure 3E, F). Thus, chemical and genetic approaches indicate that ARF6 inactivation is both necessary and sufficient to explain the ability of SH-BC-893 to boost intracellular ASO levels.

Although the ARF6 inhibitors SecinH3 and NAV2729 increased intracellular ASO levels, they did not block delivery to lysosomes like SH-BC-893 (Figure 3A, G). In contrast, the PIKfyve inhibitors YM201636 and apilimod reduced the amount of ASOs within lysosomes by 80–90% similar to SH-BC-893. However, these PIKfyve inhibitors did not increase intracellular ASO levels and had only a minimal effect on recycling (Figure 3A–D). In keeping with the model in Figure 1A, combining ARF6 and PIKfyve inhibition was necessary and sufficient to recapitulate the full effects of SH-BC-893 on ASO localization. Cells treated with both the ARF6 inhibitor NAV2729 and the PIKfyve inhibitor YM201636 had more intracellular ASOs outside of lysosomes matching the effects of SH-BC-893. Taken together, these experiments indicate that inhibiting ARF6 and PIKfyve-dependent trafficking in parallel accounts for effects of SH-BC-893 on intracellular ASO accumulation and trafficking as proposed in Figure 1A.

**Figure 4.**
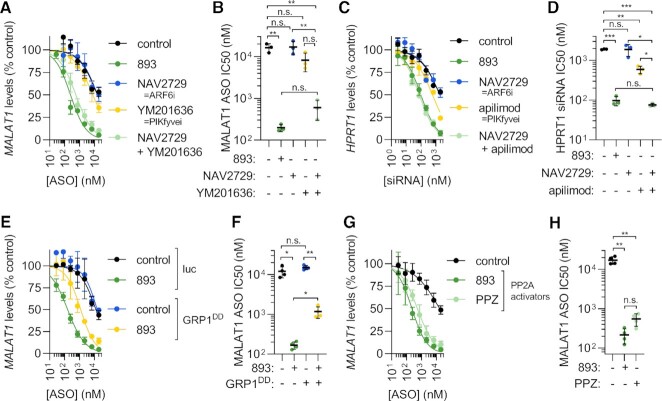
Simultaneous PIKfyve and ARF6 inhibition is both necessary and sufficient to account for the increase in ASO and siRNA activity in SH-BC-893-treated cells. (**A**) *MALAT1* levels in HeLa cells treated with the indicated concentrations of cEt gapmer ASO targeting *MALAT1* ± SH-BC-893 (5 μM), NAV2729 (12.5 μM), YM201636 (800 nM), or both for 24 h. Mean ± SD shown, *n* = 3. (**B**) IC_50_s from each biological replicate in (A); mean ± SD shown. Due to unequal SD, a Brown–Forsythe and Welch ANOVA test was used with Dunnett's T3 test to correct for multiple comparisons; **P < 0.01. (**C**) *HPRT1* mRNA levels in HeLa cells treated with the indicated concentrations of a palmitate-conjugated siRNA targeting *HPRT1* ± SH-BC-893 (5 μM), NAV2729 (12.5 μM), apilimod (100 nM) or NAV2729 and apilimod for 24 h. Mean ± SD shown, *n* = 3. (**D**) IC_50_s from each biological replicate in (C); mean ± SD shown. Using an ordinary one-way ANOVA with Sidak's multiple comparison test, **P < 0.01; ***P < 0.001. (**E**) *MALAT1* levels in HeLa cells stably expressing luciferase or GRP1^DD^ treated with the indicated concentrations of cEt ASO targeting *MALAT1* ± SH-BC-893 (5 μM) for 24 h. Mean ± SD shown, *n* = 3. (**F**) IC_50_s from each biological replicate in (E); mean ± SD shown. (**G**) *MALAT1* levels in HeLa cells treated with the cEt ASO targeting MALAT1 ± SH-BC-893 (5 μM) or PPZ (15 μM) for 24 h. Mean ± SD shown, *n* = 3. (**H**) IC50s from each biological replicate in (G); mean ± SD shown. Because SD are not equal, Brown-Forsythe and Welch ANOVA test was used with Dunnett's T3 test to correct for multiple comparisons; **P < 0.01.

To determine the extent to which increased intracellular ASO levels and extra-lysosomal localization contribute to the improved ASO activity in SH-BC-893-treated cells (Figure 2 and Supplementary Figures S4 and S5), the effects of ARF6 inhibitors and PIKfyve inhibitors alone and in combination on ASO activity were assessed by RT-qPCR. Despite increasing the amount of intracellular ASOs to a similar extent as SH-BC-893 (Figure 3A, B), the ARF6 inhibitors NAV2729 and SecinH3 failed to increase ASO activity (Figure 4A,B and Supplementary Figure S8A,B). Lack of potentiation most likely reflects ASO accumulation in lysosomes when only ARF6 is inhibited (Figure 3A, G) as escape from this site is expected to be inefficient. Although they reduced lysosomal co-localization to a similar degree as SH-BC-893 (Figure 3A,G), the PIKfyve inhibitors YM201636 or apilimod increased ASO activity by only 3–4-fold (Figure 4A, B and Supplementary Figure S8A, B). The failure of PIKfyve inhibitors to increase ASO activity to a similar extent as SH-BC-893 likely reflects their inability to increase intracellular ASO accumulation (Figure 3A-B). Combining the ARF6 inhibitor NAV2729 with the PIKfyve inhibitor YM201636 produced marked synergy, improving ASO activity to the same extent as SH-BC-893 (Figure 4A, B). Importantly, SH-BC-893, the ARF6 inhibitors, the PIKfyve inhibitors, and the combination were not toxic to cells at the concentrations that disrupt endolysosomal trafficking and modulate ASO activity (Supplementary Figure S8C). As for SH-BC-893 (Figure 2H–J), the synergy between ARF6 inhibitors and PIKfyve inhibitors was not cell type- or platform-specific. PIKfyve/ARF6 inhibitor combinations also improved the activity of a *Malat1*-targeting cEt gapmer in MEFs and a *HPRT1*-targeting siRNA in HeLa cells (Supplementary Figure S8D, E and Figure 4C, D). In sum, structurally distinct chemical inhibitors and genetic studies indicate that simultaneous ARF6 and PIKfyve inhibition synergistically improve ASO activity.

Genetic experiments confirmed that simultaneous ARF6 and PIKfyve inhibition was required for ASO potentiation. Similar to ARF6 knockdown, PIKfyve knockdown severely limited cell viability and growth. As small molecule inhibition of ARF6 or PIKfyve is well tolerated (Supplementary Figure S8C), these proteins likely have essential non-enzymatic functions in cells. As was done for ARF6 (Figure 3E, F), PIKfyve activity was modulated indirectly. PIKfyve functions as part of a heterotrimeric complex, and can be inhibited by over-expressing its scaffold VAC14 (52,53). VAC14 over-expression produced robust cytoplasmic vacuolation (Supplementary Figure S8F). Consistent with published reports that PIKfyve-dependent vacuolation is not cytotoxic (54,55), VAC14 over-expressing cells were fully viable and proliferated normally for several weeks of continuous culture despite their highly vacuolated state. Recapitulating the effects of chemical PIKfyve inhibitors (Figure 4A–B and Supplementary Figure S8A–B, D–E), VAC14 over-expression increased ASO activity by ∼2-fold (Supplementary Figure S8G, H). This result confirms that PIKfyve inhibition alone is not sufficient to promote ASO activity.

Consistent with the model that dual ARF6 and PIKfyve inactivation is required to phenocopy the effects of SH-BC-893, VAC14 over-expression synergized with the otherwise ineffective ARF6 inhibitor NAV2729 to potentiate ASO activity (Supplementary Figure S8G-H). Conversely, inhibition of ARF6-mediated endocytic recycling was necessary for SH-BC-893-mediated ASO potentiation. GRP1^DD^ expressing cells that are resistant to endocytic recycling inhibition by SH-BC-893 ((40) and Figure 3E, F) were also significantly less sensitive to the ASO-potentiating effects of SH-BC-893 (Figure 4E, F). In summary, studies with chemical inhibitors and genetic approaches both support the model that simultaneous ARF6 and PIKfyve inhibition is necessary and sufficient to account for the effects of SH-BC-893 on ASO uptake, distribution, and activity.

SH-BC-893 disrupts both endocytic recycling and lysosomal fusion by activating PP2A (Figure 1A and (39,40,42)). The dopaminergic antagonist perphenazine (PPZ) is structurally distinct from SH-BC-893 (Supplementary Figure S1) but also activates PP2A (56,57). Like SH-BC-893, PPZ inhibits ARF6-dependent endocytic recycling (40) and vacuolates cells similar to PIKfyve inhibitors (Supplementary Figure S9A). Consistent with the model that SH-BC-893’s effects on ASO trafficking and activity lie downstream of PP2A-dependent ARF6 and PIKfyve inactivation (Figure 1A), PPZ enhanced the intracellular accumulation of ASOs, blocked endocytic recycling, and increased the extralysosomal fraction of ASOs phenocopying the effects of SH-BC-893 (Supplementary Figure S9B–E). Like SH-BC-893, PPZ is also not cytotoxic under the conditions where it disrupts endolysosomal trafficking (Supplementary Figure S9F). Consistent with its effects on intracellular ASO levels and localization (Supplementary Figure S9B–E), PPZ increased ASO activity to a similar extent as SH-BC-893 (Figure 4G, H). Notably, SH-BC-893 is 3 times more potent than PPZ. As with SH-BC-893, potentiation by the PP2A activator PPZ was independent of the RNA target or cell type (Supplementary Figure S9G-I). Together, these results with molecules that are structurally unrelated to SH-BC-893, the ARF6 inhibitors NAV2729 and SecinH3, the PIKfyve inhibitors YM201636 and apilimod, and the PP2A activator PPZ, support the model that SH-BC-893 improves oligonucleotide activity via PP2A-dependent changes in ARF6- and PIKfyve-dependent endolysosomal trafficking (Figure 1A and (39,40)).

### Oral administration of SH-BC-893 safely potentiates systemically delivered ASOs in both the liver and extra-hepatic tissues

**Figure 5.**
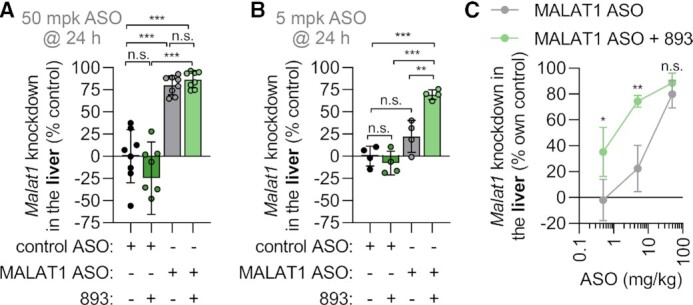
SH-BC-893 enhances activity of systemically administered ASOs in the liver. (**A**) *Malat1* knockdown in the livers of male Balbc/J mice treated with SH-BC-893 (120 mg/kg P.O.) 2 h before ASO (50 mg/kg S.C.) and sacrificed 24 h after a single dose. Non-targeting (control) or *Malat1*-targeting cEt gapmer ASO were used. Mean ± SD shown, *n* = 8. Using an ordinary one-way ANOVA with Tukey's correction for multiple comparisons, ***P < 0.001. (**B**) As in (A), except mice were given 5 mg/kg ASO and *n* = 4. (**C**) *Malat1* knockdown in mice treated as in (A) with 120 mg/kg SH-BC-893 and the indicated dose of cEt *Malat1* ASO. Mean ± SD shown, *n* = 4 except 50 mg/kg group where *n* = 8. Using an unpaired *t*-test to compare results ± SH-BC-893, *P < 0.05 and **P < 0.01. RNA levels are expressed relative to the housekeeping gene *Ppia* using the 2^−ΔΔCt^ method. In (A, B), knockdown is calculated relative to the mean from the mice receiving the non-targeting ASO and water vehicle. In (C), knockdown is expressed relative to the mean from the non-targeting ASO group for either the vehicle- or SH-BC-893-treated mice.

Inefficient oligonucleotide uptake and endosomal escape limits target engagement in all tissues, including the liver (9,10,46). Prior work established that SH-BC-893 reaches active concentrations in the liver 4 h after oral administration of 120 mg/kg (41). To determine whether SH-BC-893 potentiated ASO activity in the liver, the cEt gapmer targeting *Malat1* was administered to mice subcutaneously. *Malat1* is often targeted in proof-of-concept studies as this lncRNA is ubiquitously expressed and not essential for cellular homeostasis (58). To control for sequence-independent effects of oligonucleotides on *Malat1* expression (47), a non-targeting, control ASO was also utilized (Supplementary Table 1). As expected, 50 mg/kg of targeted but not control ASO administered subcutaneously reduced *Malat1* RNA levels in the liver by 80% (Figure 5A). At this high ASO dose, knockdown efficiency was not significantly improved by SH-BC-893. At lower ASO doses, the potentiating effect of SH-BC-893 became apparent (Figure 5B, C). With SH-BC-893 co-administration, 5 and 0.5 mg/kg Malat1-targeted ASO produced similar *Malat1* knockdown as the 50 and 5 mg/kg doses in the absence of SH-BC-893 (Figure 5A–C and Supplementary Figure S10A). Fitting a curve to the dose response shown in Figure 5C, the ED50 for knockdown was 15 mg/kg in control mice and 1 mg/kg in SH-BC-893-treated mice. Targeted delivery via GalNAc conjugation increases ASO uptake and activity in hepatocytes (59,60). As expected, the GalNAc_3_-conjugated form of the MALAT1 ASO (GN3-ASO) was already extremely potent in the liver (Supplementary Figure S10B). SH-BC-893 only modestly improved its activity. Given the different route of entry and pharmacology, a kinetic study combined with a full dose response curve would be required to fully appreciate the extent to which SH-BC-893 would further improve ASO potency for GalNAc-conjugates. In sum, SH-BC-893 increases the activity of systemically delivered ASOs in the liver.

**Figure 6.**
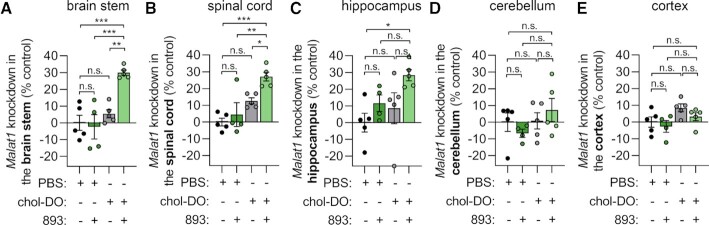
SH-BC-893 increases the activity of systemically-delivered, cholesterol-functionalized DNA/DNA duplexed oligonucleotides in the CNS. (A–E) *Malat1* knockdown in the brain stem (**A**), spinal cord (**B**), hippocampus (**C**), cerebellum (**D**) or cortex (**E**) of male Balbc/J mice treated with SH-BC-893 (120 mg/kg P.O.) 2 h before subcutaneous dosing with cholesterol-functionalized duplexed cEt gapmer targeting *Malat1* (100 mg/kg of duplex, 50 mg/kg of ASO strand S.C.). Mice received two doses 2 days apart and were sacrificed 5 days after the last dose. Mean ± SEM shown, *n* = 5. Using an ordinary one-way ANOVA with Tukey's correction for multiple comparisons, ***P < 0.001. RNA levels were expressed relative to the housekeeping gene *Ppia* using the 2^−ΔΔCt^ method and knockdown expressed relative to the mean of the group receiving both vehicles.

Orally administered SH-BC-893 also reaches active concentrations in the brain (41). Many CNS diseases, including lethal neurodegenerative diseases where there is a high unmet need, could be treated with oligonucleotide therapeutics. Because unconjugated oligonucleotides do not cross the blood brain barrier (BBB), intrathecal administration is required to access CNS targets (9,10). However, intrathecal or intracerebroventricular administration is not patient-friendly. If oligonucleotides could engage CNS targets after systemic delivery, it could allow home administration and improve provider and patient uptake. It was recently discovered that systemically-delivered oligonucleotides can cross the BBB if duplexed with a cholesterol-conjugated sense strand (61). However, multiple high doses of these duplexes are required to achieve significant knockdown in the CNS. We therefore evaluated whether SH-BC-893 could improve target RNA knockdown in the CNS of mice treated with the *Malat1* targeting cEt ASO duplexed with a cholesterol-functionalized DNA sense strand (Supplementary Table 4). Consistent with prior reports (61), two subcutaneous 50 mg/kg doses of duplexed ASOs were insufficient to produce robust *Malat1* knockdown in CNS tissues (Figure 6A–E). However, co-administration with SH-BC-893 enabled statistically significant knockdown of ∼30% in the brain stem and spinal cord with a promising trend in the hippocampus and cerebellum. No knockdown was observed in the cortex in control or 893-treated mice. The lack of activity in the cortex could be due to low penetration of ASOs in this region as SH-BC-893 has been shown to reach active concentrations in the cortex (41). These results clearly demonstrate that SH-BC-893 can improve oligonucleotide activity in at least a subset of CNS tissues.

**Figure 7.**
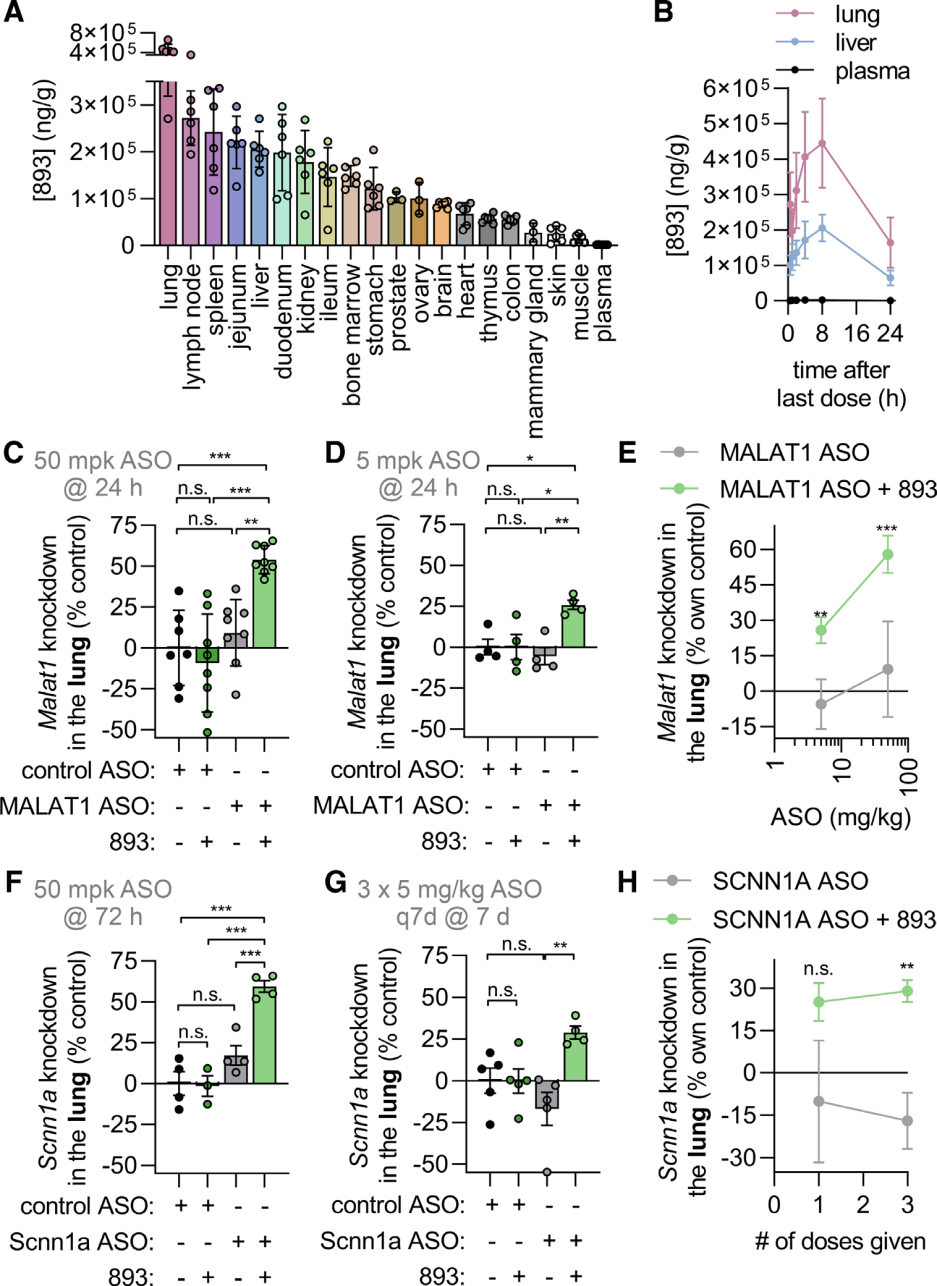
SH-BC-893 sensitizes the lung to systemically administered ASOs. (**A**) Tissue SH-BC-893 levels in male (*n* = 3) or female (*n* = 3) CD1 mice treated with 120 mg/kg P.O. Q.D. for 5 days and sacrificed 8 h after the last dose. Mean ± SD shown, *n* = 6. (**B**) As in (A) but in mice sacrificed at the indicated time points after the last dose. (**C**) *Malat1* knockdown in the lungs of male Balbc/J mice treated with SH-BC-893 (120 mg/kg P.O.) 2 h before ASO (50 mg/kg S.C.) and sacrificed 24 h after a single dose. Non-targeting (control) or *Malat1*-targeting cEt gapmer ASO were used. Mean ± SD shown, *n* = 8. Using an ordinary one-way ANOVA with Tukey's correction for multiple comparisons, ***P < 0.001. (**D**) As in (C), except mice were given 5 mg/kg ASO and *n* = 4. (**E**) *Malat1* knockdown in mice treated as in (C) with 120 mg/kg SH-BC-893 and the indicated dose of cEt *Malat1* ASO. Mean ± SD shown, *n* = 4 except 50 mg/kg group where *n* = 8. Using an unpaired *t*-test to compare results ± SH-BC-893, *P < 0.05 and **P < 0.01. (**F**) *Scnn1a* (aka ENaCα) knockdown in the lungs of male Balbc/J mice treated with SH-BC-893 (120 mg/kg P.O.) 2 h before ASO (50 mg/kg S.C.) and sacrificed 72 h after a single dose. Non-targeting (control) or *Scnn1a*-targeting cEt gapmers was used. Mean ± SD shown, *n* = 4. Using a one-way ANOVA with Tukey's correction for multiple comparisons, ***P < 0.001. (**G**) As in (F), except three doses of 5 mg/kg ASO were given at 7 days intervals and mice sacrificed 7 days after the last dose. (**H**) As in (G), except expressed as a function of number of doses received and normalized to the non-targeting ASO control. Using an unpaired *t*-test to compare results ± SH-BC-893, **P < 0.01. RNA levels are expressed relative to the housekeeping gene *Ppia* using the 2^−ΔΔCt^ method. In (C, D, F, G), knockdown is calculated relative to the mean from the mice receiving the non-targeting ASO and water vehicle. In (E, H), knockdown is expressed relative to the mean from the non-targeting ASO group for either the vehicle- or SH-BC-893-treated mice.

To predict which additional extrahepatic tissues might be susceptible to SH-BC-893-mediated ASO potentiation, SH-BC-893 levels were determined in a variety of organs after oral administration (Figure 7A). SH-BC-893 levels were highest in the lung (Figure 7A, B), a tissue that is basally resistant to systemically administered ASOs due to limited ASO accumulation at this site (62,63). As expected, a single 50 or 5 mg/kg subcutaneous dose of ASO did not produce knockdown in the lung (Figure 7C, D). In contrast, oral administration of SH-BC-893 enabled ASO-dependent reductions in lung *Malat1* RNA levels of 54% or 26% 24 h after a single 50 or 5 mg/kg subcutaneous ASO dose, respectively. Potentiation by SH-BC-893 was similarly robust when measured 3 d after dosing (Supplementary Figure S10C). Although ASOs and SH-BC-893 are both present at reasonably high levels in the kidney and spleen ((64) and Figure 7A and Supplementary Figure S10D), no potentiation was observed in these tissues (Supplementary Figure S10E–I). The lack of potentiation could reflect different limitations on ASO delivery in different tissues, accumulation of ASO and SH-BC-893 in different cell types, and/or the fact that tissue-level LC-MS/MS measurements fail to discriminate between intracellular and extracellular ASOs and/or SH-BC-893. Statistically significant ASO potentiation was not observed in tissues with low SH-BC-893 levels such as skeletal and heart muscle, although a promising trend was observed in the quadriceps that might be improved with repeated dosing (Figure 7A and Supplementary Figure S10J, K). In summary, co-administration of SH-BC-893 rendered systemically administered ASO active in the lung.

Potentiating effects in the lung were intriguing as oligonucleotide therapeutics are being developed to treat lung diseases (62,65,66). An ASO targeting the *Scnn1a* mRNA that encodes a subunit of the ENaC sodium channel has been tested in cystic fibrosis patients and may also have benefits in other lung diseases characterized by mucus dehydration such as chronic obstructive pulmonary disease (COPD) (65–67). SH-BC-893-dependent potentiation may vary with different ASO sequences and targets. Because the MALAT1 ASO widely used in proof-of-concept studies is 2–3-fold more potent than most other cEt gapmers, other oligonucleotides may exhibit less potentiation with SH-BC-893. Additionally, individual cell types present within a tissue may be differentially sensitive to SH-BC-893-dependent potentiation (62–64). For these reasons, SH-BC-893 was also tested with a cEt gapmer ASO targeting the *Scnn1a* mRNA expressed in lung epithelial cells. At the 50 mg/kg ASO dose, SH-BC-893 improved *Scnn1a* knockdown from 17% to 59% (Figure 7F). While ENaC is also expressed in the kidney, statistically significant knockdown was not observed with subcutaneous administration of the Scnn1a ASO even in mice treated with SH-BC-893 (Supplementary Figure S10L). Although knockdown did not achieve statistical significance after a single 5 mg/kg dose of Scnn1a ASO and SH-BC-893, 30% knockdown was achieved after repeat dosing with the combination while Scnn1a ASO alone was ineffective (Figure 7G, H and Supplementary Figure S10M). Thus, SH-BC-893 improves ASO activity in lung epithelial cells that are targeted by oligonucleotide therapeutics designed to treat cystic fibrosis. In sum, the results presented here establish the feasibility of improving therapeutic oligonucleotide delivery to both hepatic and extrahepatic tissues by co-administering small molecules like SH-BC-893 that block endocytic recycling and lysosomal fusion.

In contrast to the historical toxicity problems associated with endolytic agents, even chronic daily administration of SH-BC-893 is well tolerated (39). After 11 weeks of daily oral administration of the effective dose of SH-BC-893 (120 mg/kg), blood chemistry revealed no signs of organ toxicity. Rapidly proliferating cells in the bone marrow and intestinal crypts were not compromised based on normal complete blood counts and grossly normal histopathology. In fact, oral administration of 120 mg/kg SH-BC-893 every other day promotes metabolic homeostasis in mice maintained on a high fat diet (41). In this study, mice provided with a running wheel engaged in the same amount of voluntary exercise whether they were treated with vehicle or with 120 mg/kg SH-BC-893 PO over the 4-week study. Voluntary wheel running is a holistic and sensitive measure of animal health (68). Together, these studies clearly establish the safety of chronic administration of the effective dose of SH-BC-893.

To confirm that SH-BC-893 did not induce acute toxicity that might have been missed in the published long-term studies, we evaluated blood chemistry in mice 24 h after treatment with vehicle, 120 or 240 mg/kg SH-BC-893 (Supplementary Figure S11C). Neither the effective dose (120 mg/kg) nor twice the effective dose (240 mg/kg) disrupted blood chemistry values, indicating that SH-BC-893 is not acutely toxic to the liver, kidney, or muscle. Consistent with the bloodwork, no significant changes in body weight were noted in mice that were maintained for more than 24 h after administration of SH-BC-893 and oligonucleotide (Supplementary Figure S11D-F). When used as an oligonucleotide potentiator, the safety margin would be further enhanced compared to published studies with repeat dosing because SH-BC-893 would only be administered as frequently as the oligonucleotide (weekly, monthly or even every 6 months) (16). Taken together, the data presented here and in published studies clearly establish that SH-BC-893 is not toxic to mice at the effective dose.

## DISCUSSION

Here, we demonstrate that the small molecule SH-BC-893 increases the activity of ASOs and siRNAs by up to 100-fold in multiple cell types without lysing endosomes. Rather than disrupting membranes, SH-BC-893 traps oligonucleotides in a pre-lysosomal compartment where they likely escape when fission and fusion reactions deform the lipid bilayer increasing permeability (27,29–33). Consistent with this mechanism of action and the well-established low permeability of the lysosomal membrane, SH-BC-893 cannot increase oligonucleotide activity if it is added after ASOs have reached lysosomes (Supplementary Figure S6C, D). Genetic approaches and multiple, structurally distinct chemical inhibitors confirmed that simultaneous inhibition of ARF6-dependent endocytic recycling and PIKfyve-dependent lysosomal fusion was necessary and sufficient for SH-BC-893 to boost intracellular ASO levels, increase accumulation of ASOs within extra-lysosomal compartments, and enhance oligonucleotide activity (Figures 3 and 4 and Supplementary Figure S8). As single agents, ARF6 inhibitors increase intracellular ASO levels but fail to potentiate ASO activity because ASOs still end up in lysosomes (Figure 3A–B, G and Figure 4B and Supplementary Figure S8A–B, D–E). Similarly, inactivating the PIKfyve-dependent lysosomal fusion pathway in isolation fails to block recycling and produces only a 2–4-fold increase in oligonucleotide activity similar to what can be achieved with previously reported small molecule potentiators (Figures 3C–D and 4A–D and Supplementary Figures S6G–H and S8A–B, D–H). In contrast, the synergistic effects of blocking recycling out of the cell and preventing ASO transit to lysosomes allows SH-BC-893 to dramatically increase oligonucleotide activity (Figures 3 and 4 and Supplementary Figure S8). SH-BC-893 is distinct from previously identified small molecule potentiators in that it robustly increases oligonucleotide activity without lysing endosomes, avoiding the toxic consequences that have prevented endolytic agents from advancing to the clinic.

Agents like SH-BC-893 may have some utility for liver-targeted ASOs. The 15-fold increase in ASO activity observed in the livers of mice treated with SH-BC-893 (Figure 5C) is on par with the potency increase reported with GalNAc conjugation, a clinically validated means to improve oligonucleotide delivery to hepatocytes (9,59). Thus, for liver targets, SH-BC-893 might offer greater access to cell types that do not express the ASGPR or offer an alternative approach to GalNAc conjugation. SH-BC-893 slightly enhanced the activity of the already quite potent GalNAc_3_-MALAT1 ASO (Supplementary Figure S10B). Additional studies with GalNAc-conjugates and other targeted oligonucleotides would be required to fully assess the potential of SH-BC-893 to improve ligand-conjugated oligonucleotide uptake in the liver and extrahepatic tissues (13,69,70).

Agents that act like SH-BC-893 are poised to render new targets in extrahepatic tissues accessible even without ligand-receptor targeting strategies. In the CNS, SH-BC-893 increased the activity of cholesterol-functionalized ASO duplexes in the brainstem and spinal cord (Figure 6A, B) with positive trends noted in the hippocampus and cerebellum (Figure 6C, D). The most sensitive extrahepatic tissue was the lung (Figure 7C–E) likely due to the accumulation of SH-BC-893 in this tissue (Figure 7A, B). Interestingly, although both ASOs and SH-BC-893 accumulate in the kidney and spleen (Figure 7A, Supplementary Figure S10D, and (64)), potentiation was limited or absent in these tissues (Supplementary Figure S10E–I, L). These results could be explained by lower basal levels of ARF6-dependent ASO recycling, reduced PIKfyve-dependent lysosomal fusion, and/or differences in the endocytic trafficking pathways used for oligonucleotide entry in these tissues. Alternatively, SH-BC-893 and ASOs may not accumulate in the same cell types in these organs; ASOs accumulate primarily in proximal tubular epithelial cells in the kidney and endothelial cells in the spleen. In heart and skeletal muscle, limited potentiation is likely explained by the low concentrations of both SH-BC-893 and ASOs in these tissues ((63,64) and Supplementary Figure S10D, J–K). Given the ubiquitous expression of PP2A, ARF6 and PIKfyve, additional tissues with SH-BC-893 levels higher than the brain (Figure 7A) may also be sensitive to potentiation. The results reported here offer an important proof of concept, but additional studies will be required to produce a comprehensive inventory of the tissues and cell types that are responsive to SH-BC-893-mediated potentiation with single or repeat oligonucleotide dosing.

While this study utilized systemic ASO delivery, SH-BC-893 should also improve the efficacy of locally administered oligonucleotide therapeutics. Administration of ASOs directly at the site of action (e.g. intravitreal, intrathecal, or aerosol delivery) increases uptake by elevating local concentrations but does not address the negative effects of ARF6-dependent recycling or PIKfyve-dependent lysosomal fusion on delivery (Figure 1A). Local administration of SH-BC-893 along with the oligonucleotide might additionally overcome any limitations imposed by the tissue pharmacokinetics of SH-BC-893. In some cases, local delivery of oligonucleotide is preferred to limit target engagement to specific tissues. For example, in cystic fibrosis therapy it is desirable to reduce ENaC levels in the lung while leaving ENaC expression in the kidney unaffected (71). Notably, SH-BC-893 increased ENaC knockdown in the lung (Figure 7F-H) but not in the kidney (Supplementary Figure S10L). Formulation work would be required to evaluate whether SH-BC-893 could be administered intrathecally or via inhalation along with the oligonucleotide, but local delivery of oligonucleotide and/or SH-BC-893 might further extend the clinical reach of small molecule-mediated potentiation.

It is also worth noting that SH-BC-893 is unlikely to diminish any long-term activity that might be sustained by slow leak of oligonucleotides from lysosomes (16). A single dose of SH-BC-893 administered with oligonucleotides would be cleared from the body in 24–48 h releasing the trafficking block and allowing trapped oligonucleotides to continue their progress through the endocytic pathway. Long-term activity likely tracks with the level of initial oligonucleotide uptake by target cells, and thus SH-BC-893 may improve knockdown duration by increasing the intracellular amount of oligonucleotide (Figures 1D–E and 3A–B and Supplementary Figure S2B, D, E, G). In summary, SH-BC-893 is likely to be compatible with other approaches that improve extrahepatic oligonucleotide delivery.

The in vivo results with SH-BC-893 are noteworthy both due to the significant gain in oligonucleotide activity and the surprising tolerability of this compound despite its profound effects on endolysosomal trafficking (39,41). SH-BC-893 does not harm even rapidly proliferating normal tissues after chronic administration of the effective dose; bone marrow suppression was not observed and rapidly dividing intestinal crypts were not compromised (39). In fact, SH-BC-893 promotes metabolic homeostasis in mice, and treated animals engage in normal levels of voluntary exercise, a holistic measure of animal health (41,68). When used as an oligonucleotide delivery potentiator, the safety margin would be further increased by infrequent dosing. Consistent with these earlier studies, acute administration of even twice the effective dose of SH-BC-893 produced no detectable changes in liver, kidney, or muscle function by blood chemistry analysis (Supplementary Figure S11C).

The tolerability of SH-BC-893 likely stems from its origin as a synthetic analog of the natural sphingolipid, phytosphingosine, that promotes survival in yeast under stress (72). This sphingolipid-responsive signaling pathway is conserved in mammalian cells suggesting that it was fine-tuned by evolution to modulate intracellular trafficking in a manner that preserves cell viability and tissue homeostasis (39–42,73). Consistent with this hypothesis, SH-BC-893 selectively kills cancer cells while sparing non-transformed cells (39). Oncogenic mutations make cancer cells constitutively anabolic and therefore hypersensitive to nutrient limitation. Indeed, this liability provides the therapeutic index for many standard-of-care cancer therapies (74). While cancer cells starve to death secondary to SH-BC-893-induced changes in endolysosomal trafficking, normal cells adapt to the moderate nutrient restriction by reducing their energy demands (39). In summary, the low toxicity of the anti-neoplastic agent SH-BC-893 in non-transformed cells and tissues likely augurs well for its use in combination with oligonucleotide therapeutics.

Going forward, oligonucleotide therapeutics will need to compete with next-generation small molecules that are also capable of hitting what were previously ‘undruggable’ targets (75). A potentiator like SH-BC-893 that permits at-home oligonucleotide administration could improve the ability of oligonucleotide therapeutics to compete with new orally administered small molecule alternatives (76). By lowering the required dose, a small molecule potentiator like SH-BC-893 could make expensive oligonucleotide therapeutics accessible to more patients. Given the established pre-clinical activities of SH-BC-893 as a single agent in cancer and obesity models (39,41), oligonucleotide therapeutics targeting these diseases might be prioritized for assessing the therapeutic value of combination therapy. For example, several oligonucleotides that entered cancer trials failed to reach efficacy benchmarks. SH-BC-893 slows autochthonous prostate tumor growth (39) and could be even more effective in combination with relevant oncology ASOs (7,77). Given its accumulation in the lung (Figure 7A), primary or metastatic lung tumors might also be responsive to an SH-BC-893/ASO combination targeting KRAS (6). The oligonucleotide-independent actions of SH-BC-893 on mitochondrial dynamics (41) may complement the activities of oligonucleotides designed to treat cancer (78), non-alcoholic steatohepatitis (NASH) (79), and/or neurodegenerative diseases (9,41,80). In conclusion, the proof-of-concept studies presented here provide a strong rationale for future work exploring the therapeutic value and safety of SH-BC-893 and/or related small molecules as oligonucleotide potentiating agents.

## DATA AVAILABILITY

All data generated and analyzed in this study are included in the manuscript and supplementary data.

## Supplementary Material

gkad023_Supplemental_File

## Notes

Present address: Punit P. Seth. Alnylam Pharmaceuticals, Cambridge, MA, USA.
