# Supplementary material for: Simultaneous inhibition of endocytic recycling and lysosomal fusion sensitizes cells and tissues to oligonucleotide therapeutics
Source: Nucleic Acids Res. 2023 Feb 2;51(4):1583–99. doi: 10.1093/nar/gkad023 (PMC9976930; doi:10.1093/nar/gkad023)
Supplement: gkad023_Supplemental_File [file gkad023_supplemental_file.pdf]

Supplementary Table 1:

Summary of ASOs used in this study.

Target	Description of chemistry	Sequence (5' → 3')
MALAT1 (mouse & human)	3-10-3 cEt gapmer	**GCA**TTCTAATAGC**AGC**
MALAT1 (mouse & human)	6-FAM-tagged 3-10-3 cEt gapmer for fluorescence microscopy	**6-FAM-GCA**TTCTAATAGC**AGC**
MALAT1 (mouse & human)	5-10-5 2'MOE gapmer	**CCAGG**CTGGTTATGA**CTCAG**
MALAT1 (mouse & human)	6-FAM-tagged 5-10-5 2'MOE gapmer for fluorescence microscopy	**6-FAM-CCAGG**CTGGTTATGA**CTCAG**
ACTN1 (human)	3-10-3 cEt gapmer	**TAA**CTTTGTGCTT**GGT**
MALAT1 (mouse & human)	5'tagged GalNAc$_3$ (GN3) tagged 3-10-3 cEt gapmer	**GN3-GCA**TTCTAATAGC**AGC**
Scnn1a (mouse)	3-10-3 cEt gapmer	**GAG**CATCTAATAC**AGC**
non-targeting	3-10-3 cEt gapmer control ASO	**CGC**CGATAAGGTA**CAC**

All gapmers contain ten 2'-deoxynucleosides flanked by either three **2',4'-constrained ethyl (cEt)** modified or five **2'-O-methoxyethyl (2'MOE)** modified riboses on the 5' and 3' ends. All cytosine residues throughout the modified oligonucleotide are 5-methyl cytosines. The internucleoside linkages are all phosphorothioate (PS). Some oligonucleotides are tagged on the 5' end with a **6-carboxyfluorescein (6-FAM)**.

Supplementary Table 2:

Primers and Probes used in this study for qRT-PCR:

target	Primers (5' → 3')	Taqman Probe (5' → 3') 6-FAM on 5' end TAMRA on 3' end
MALAT1 (human)	Fwd: AGGCGTTGTGCGTAGAGGAT<br>Rev: AAAGGTTACCATAAGTAAGTTCCAGAAAA	AGTGGTTGGTAAAAATCCGTGAGGTCGG
Malat1 (mouse)	Fwd: TGGGTTAGAGAAGGCGTGTACTG<br>Rev: TCAGCGGCAACTGGGAAA	CGTTGGCACGACACCTTCAGGGACT
ACTN1 (human)	Fwd: CAATATGGCGGGCACCAA<br>Rev: GGTCCCATTTGCCATTGATC	CCCTACACAACCATCACGCCTCAGG
Ppia (mouse)	Fwd: TCGCCGCTTGCTGCA<br>Rev: ATCGGCCGTGATGTCGA	CCATGGTCAACCCCACCGTGTTC
Scnn1a (mouse)	Fwd: GGTGTAGAGTTCTGTGACTACC<br>Rev: AGCTTGTAGTTGGTCACACTG	CTGCCTTCTCCTTGGATAGCCTGG
ACTB (human)	Fwd: CGGACTATGACTTAGTTGCGTTACA<br>Rev: GCCATGCCAATCTCATCTTGT	CCTTTCTTGACAAAACCTAACTTGCGCAGA

target	Taqman Assay Primer/Probe Mix from Applied Biosystems
HPRT1 (human)	Taqman Assay ID: Hs99999909_m1<br>Catalog #: 4333768F

Supplementary Table 3:

Summary of siRNAs used in this study.

Target	Sequence & Chemistry
HPRT1 (human)	5′-pAsUsAAAAUCUACAGUCAUAGGAsAsU-3′<br>3′-UsAsUUUUAGAUGUCAGUAUCCU-5′-C_{16}-palmitate<br>2'-OMe 2'-F-RNA s = phosphorothioate p = 5'-phosphate

Red = 2'-O-methyl (2'-OMe) modified riboses
Green = 2'fluorine (2'-F-RNA) modified riboses
s = phosphorothioate (PS) internucleoside linkage

Supplementary Table 4:

Description of cholesterol-functionalized DNA/DNA duplexed oligos

Target	Sequence & Chemistry
MALAT1 (human & mouse)	5'GsCsAsTsTsCsTsAsAsTsAsGsCsAsGsC3'<br>3'CsGsU A A G A T T A T C G UsCsG5'-TEG-cholesterol

Blue = 2',4'-constrained ethyl (cEt)
Yellow = 2'fluorine (2'-F-RNA) modified riboses
s = phosphorothioate (PS) internucleoside linkage

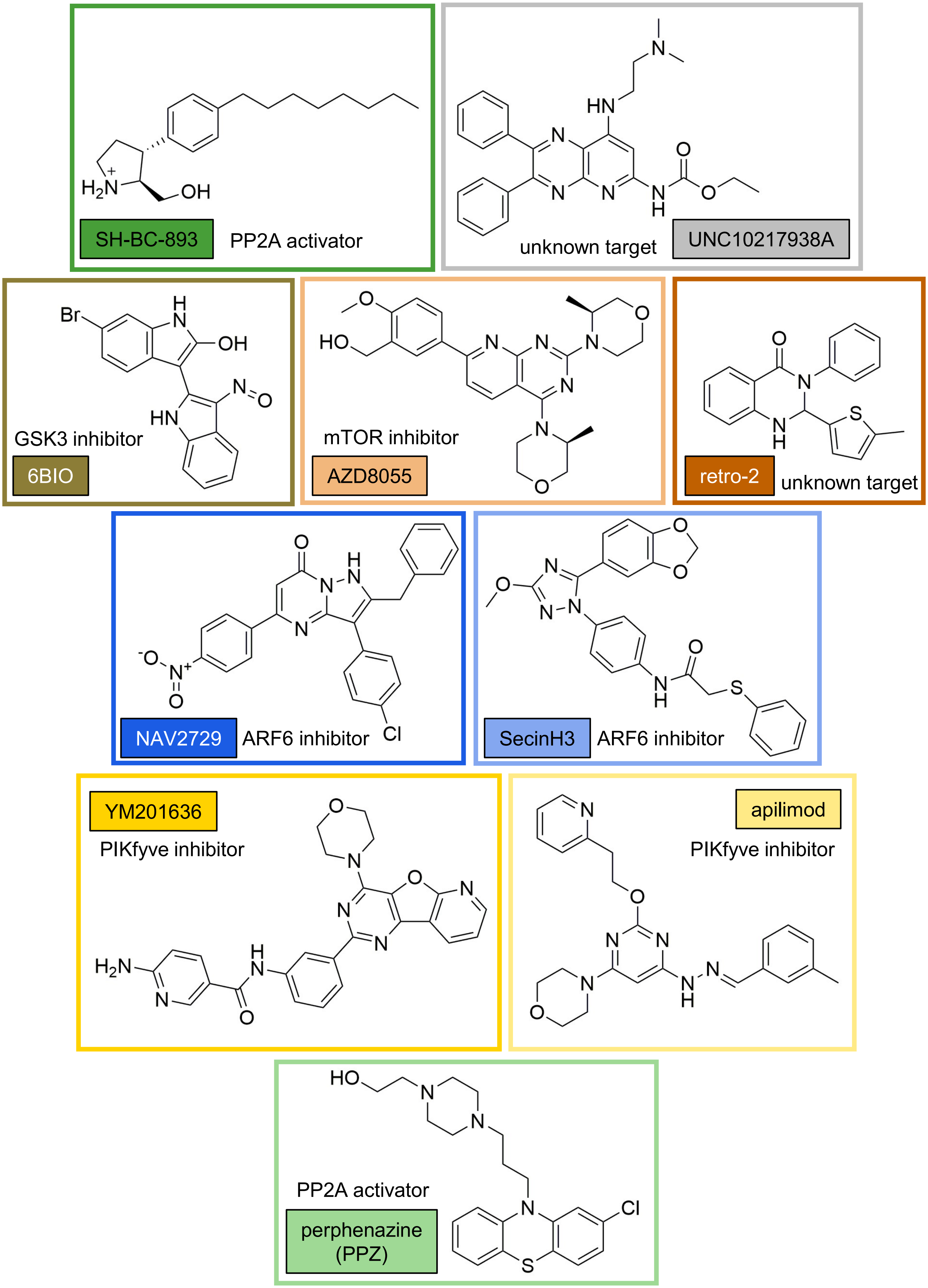

Supplementary Fig. S1: Chemical structures of all small molecules used in this study. Molecular targets are indicated.

Supplementary Fig. S2

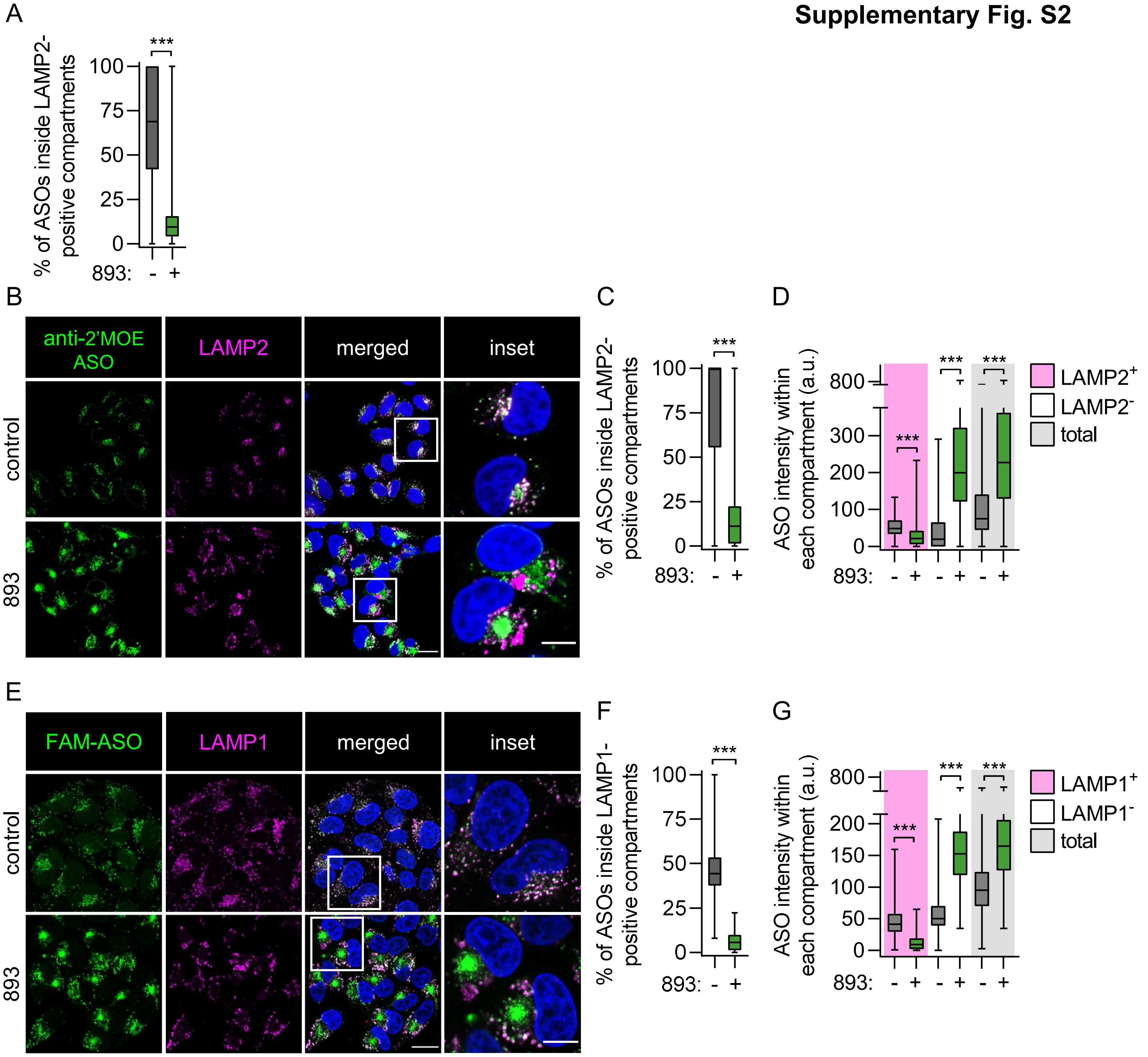

Supplementary Fig. S2: SH-BC-893 increases ASO delivery to extra-lysosomal compartments. **(A)** Quantification of the images in (Fig. 1B). Percent of the total intracellular ASO that is within LAMP2-positive lysosomes is quantified. **(B)** HeLa cells treated with an untagged 5-10-5 2'MOE gapmer (2 µM) ± SH-BC-893 (5 µM) for 6 h then stained with antibodies for PS-ASOs and endogenous LAMP2. Scale bar = 20 µm. **(C)** Quantification of the images in (B). Percent of the total intracellular ASO that is within LAMP2-positive lysosomes is quantified. **(D)** Quantification of the raw intensity values for ASO from images in (B) within LAMP2-positive, LAMP2-negative, and total cellular areas. At least 100 cells were quantified from 2 independent experiments. Using a Mann-Whitney t test to correct for data that is not normally distributed, ***, $p<0.001$. **(E-G)** As in (B-D), except FAM-tagged 3-10-3 cEt gapmer used and lysosomes marked with antibodies to endogenous LAMP1. Scale bar = 20 µm.

Supplementary Fig. S3

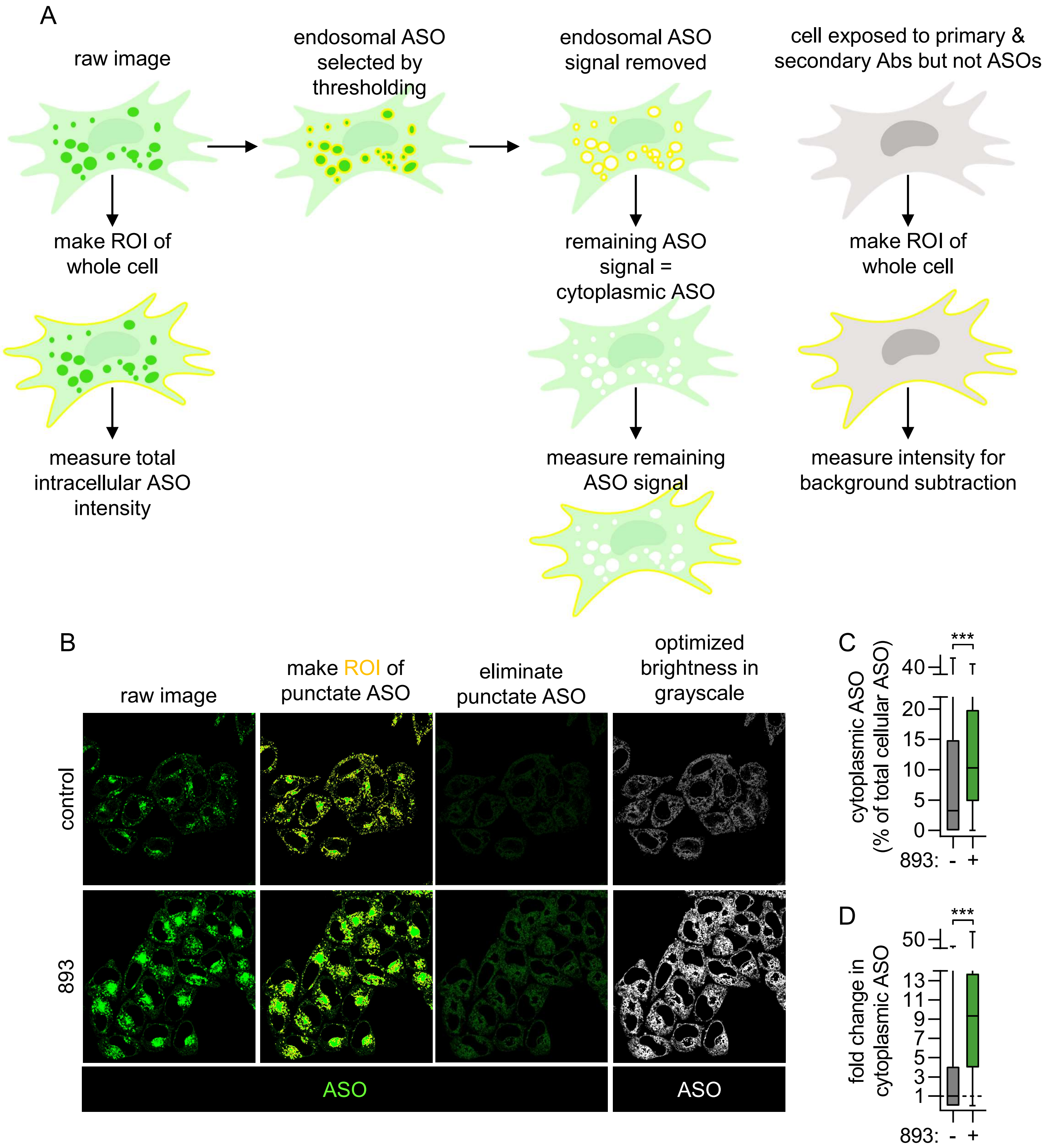

Supplementary Fig. S3: SH-BC-893 increases cytoplasmic ASO levels. (A) Figure depicting method to measure cytoplasmic ASO levels. In brief, endosomal ASO signal was eliminated by generating regions of interest (ROIs) on thresholded images, leaving the diffuse cytoplasmic signal remaining. Background elimination was performed by quantifying fluorescent signal in cells that were not exposed to ASOs but stained with both primary and secondary antibodies. **(B)** HeLa cells treated with a 3-10-3 cEt ASO targeting *MALAT1* (2 μM) ± SH-BC-893 (5 μM) for 6 h and stained with antibodies to PS-ASOs. Scale bar = 20 μm. For inset, scale bar = 10 μm. Showing multiple steps of the quantification method described in A. **(C-D)** Quantification of (B), showing percent of total intracellular ASOs that is cytoplasmic (C) or the total raw signal of ASOs in the cytoplasm (D). Using a Mann-Whitney t test to correct for data that is not normally distributed, ***, $p<0.001$.

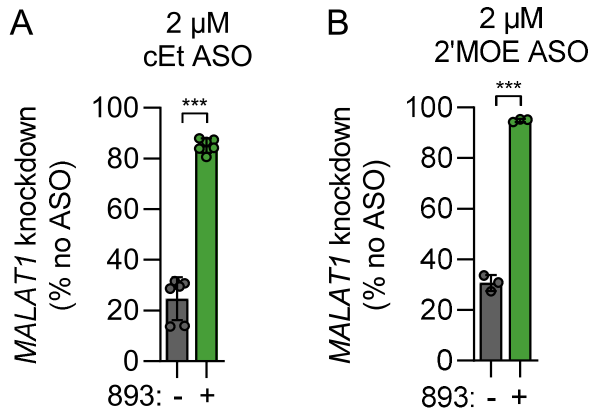

Supplementary Fig. S4: SH-BC-893 increases ASO activity. (A) *MALAT1* knockdown in HeLa cells treated with a 3-10-3 cEt gapmer (2 µM) targeting *MALAT1* ± SH-BC-893 (5 µM) for 24 h. Mean ± SD shown, n=6. Using an unpaired two-tailed t test, ***, p<0.001. **(B)** As in (A), except with a 5-10-5 2'MOE gapmer, n=3.

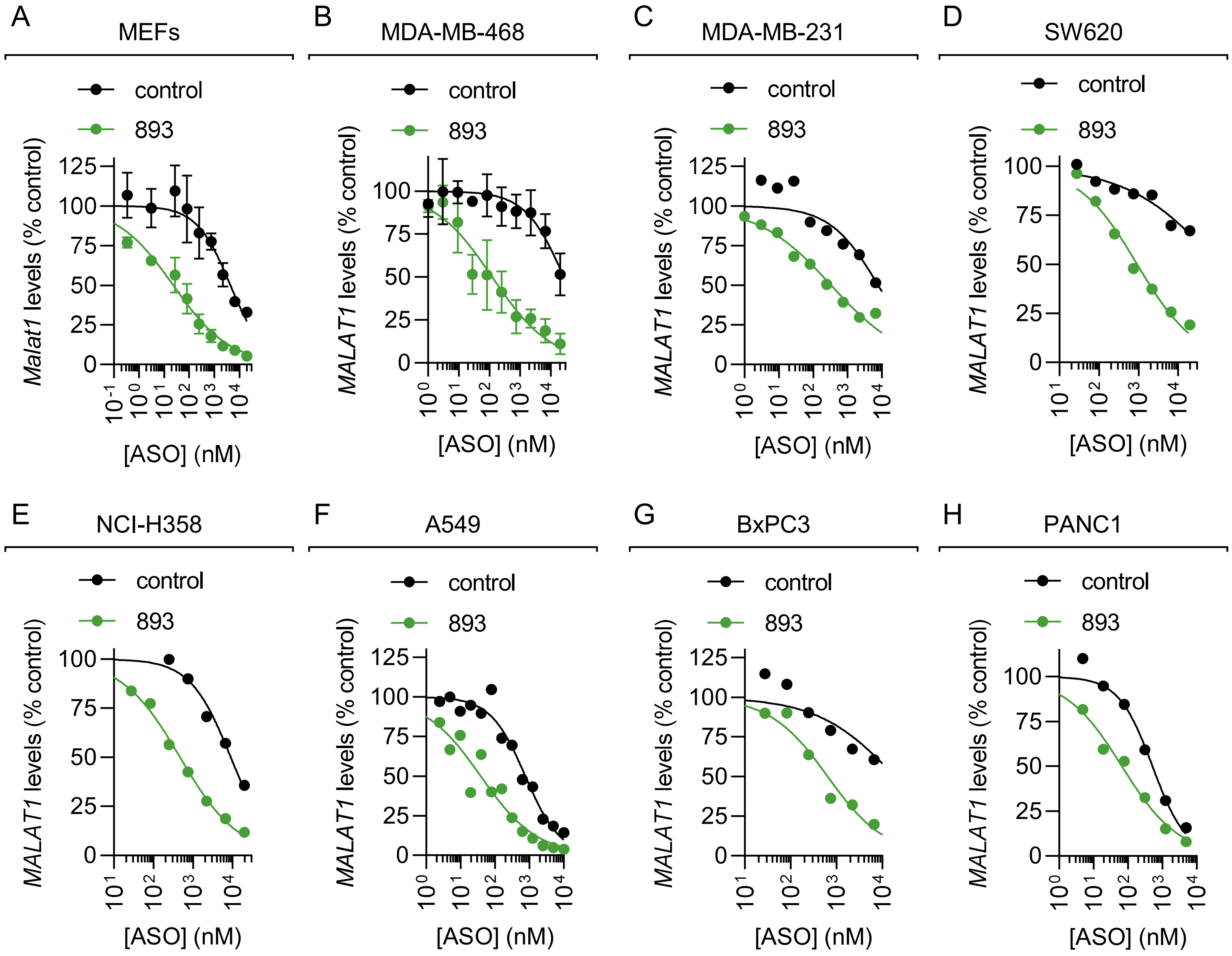

Supplementary Fig. S5: Individual graphs for the IC50s presented in Fig. 2H. (A) *Malat1* levels in MEFs treated with a 3-10-3 cEt gapmer targeting *Malat1* ± SH-BC-893 (5 μM) for 24 h. Mean ± SD shown, n=3. **(B-H)** As in (A), except MDA-MB-468 cells (B), MDA-MB-231 cells & n=1 (C), SW620 cells & n=1 (D), NCI-H358 cells & n=1 (E), A549 cells & n=2 (F), BxPC3 cells & n=1 (G), and PANC1 cells & n=1 (H).

Supplementary Fig. S6

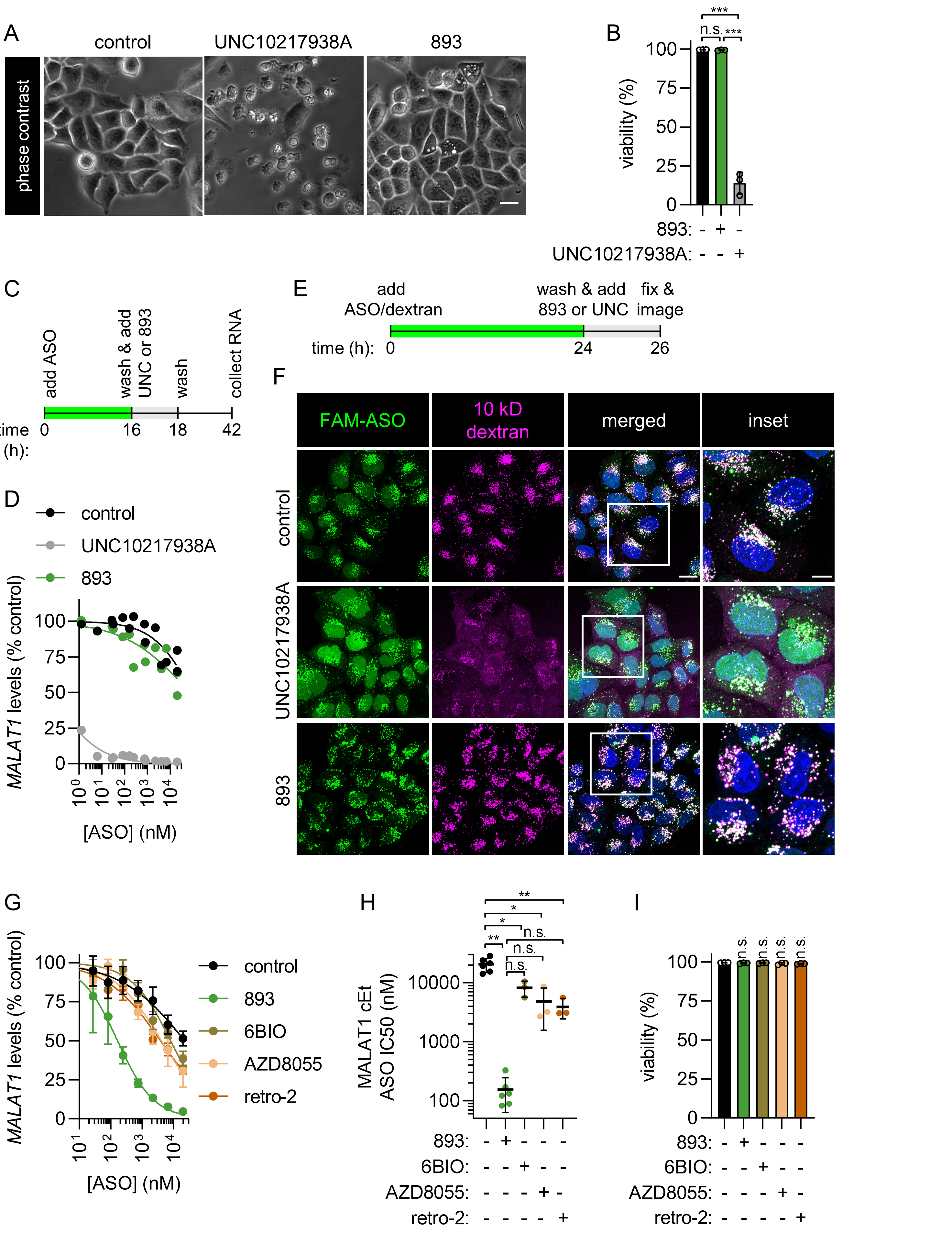

Supplementary Fig. S6: SH-BC-893 is distinct from endolytic agents and more effective than previously identified oligonucleotide-potentiating small molecules. (A) Phase contrast microscopy of HeLa cells treated with UNC10217938A (10 µM) or SH-BC-893 (5 µM) for 24 h. Scale bar = 20 µm. **(B)** Viability measured by vital dye (DAPI) exclusion through flow cytometry analysis in HeLa cells treated as in (A). Using an ordinary one-way ANOVA with Tukey's correction for multiple comparisons. Mean ± SD shown, n=3. **(C)** Timeline for the published experiments performed evaluating UNC10217938A's ability to potentiate ASO. **(D)** *MALAT1* levels in HeLa cells treated with the indicated concentrations of 3-10-3 cEt gapmer targeting *MALAT1* ± SH-BC-893 (5 µM) or UNC10217938A (10 µM) using the timeline in (C). **(E)** Timeline for (F). **(F)** HeLa cells loaded with a FAM-tagged 3-10-3 cEt gapmer (2 µM) and Alexa Fluor 594-tagged 10 kD dextran (200 µg/ml) for 24 h before treating with UNC10217938A (10 µM) or SH-BC-893 (5 µM) for 2 h before fixing and imaging by confocal microscopy. Scale bar = 20 µm. For inset, scale bar = 10 µm. n.s. = not significant. **(G)** *MALAT1* levels in HeLa cells treated with the indicated concentrations of 3-10-3 cEt gapmer targeting *MALAT1* ± SH-BC-893 (5 µM), 6BIO (3 µM), AZD8055 (500 nM), or retro-2 (100 µM) for 24 h. Mean ± SD shown, n=3-6. **(H)** IC50s from each biological replicate in (G); mean ± SD shown. Due to unequal SD, a Brown-Forsythe and Welch ANOVA test was used with Dunnett's T3 test to correct for multiple comparisons; **, $p<0.01$, *, $p<0.05$. **(I)** Viability measured by vital dye (DAPI) exclusion through flow cytometry analysis in HeLa cells treated as in (G). Using an ordinary one-way ANOVA with Tukey's correction for multiple comparisons. Mean ± SD shown, n=3.

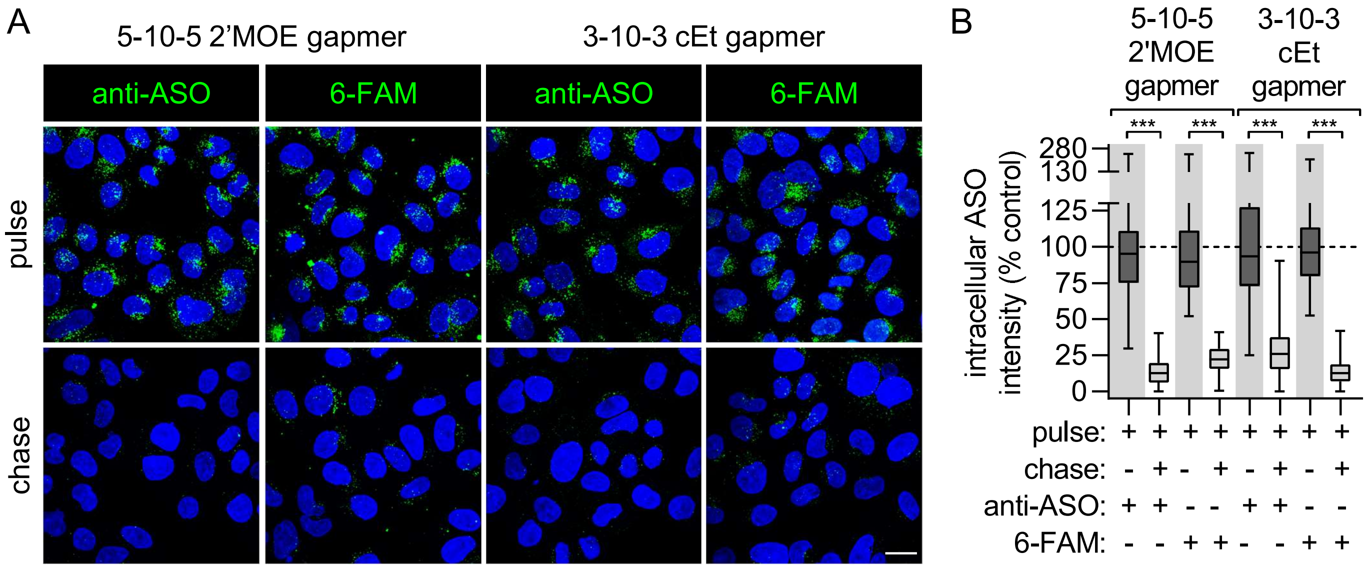

Supplementary Fig. S7: The loss of ASO signal during the chase is not due to a quenching effect on the 6-FAM fluorophore. (A) HeLa cells were pulsed with untagged or 6-FAM-tagged 5-10-5 2'MOE or 3-10-3 cEt gapmers (2 µM) for 1 h, washed, and then chased in media lacking ASOs for 2 h prior to staining with antibodies for PS-ASOs (for untagged ASOs) or imaging directly (for 6-FAM-tagged ASOs). Scale bar = 20 µm. **(B)** Quantification of the intracellular ASO fluorescence of cells in (A). At least 80 cells were quantified from 1 experiment. Because data is not normally distributed, a Kruskal-Wallis ANOVA was used with Dunn's test to correct for multiple comparisons. ***, p<0.001.

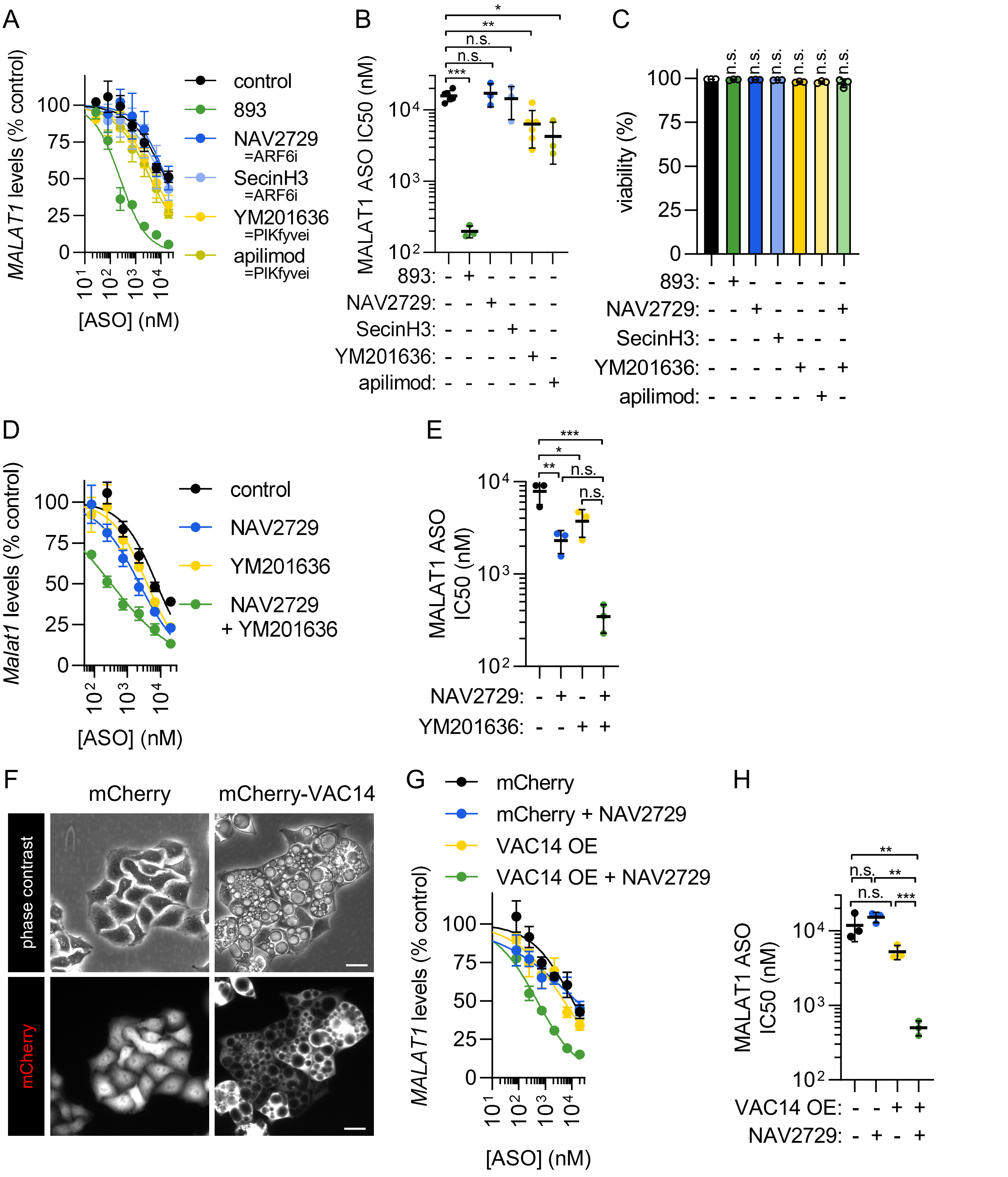
A
MALAT1 levels (% control)
[ASO] (nM)
control
893
NAV2729
=ARF6i
SecinH3
=ARF6i
YM201636
=PIKfyvei
apilimod
=PIKfyvei
B
MALAT1 ASO IC50 (nM)
893:
NAV2729:
SecinH3:
YM201636:
apilimod:
n.s.
C
viability (%)
n.s.
893:
NAV2729:
SecinH3:
YM201636:
apilimod:
D
Malat1 levels (% control)
[ASO] (nM)
control
NAV2729
YM201636
NAV2729
+ YM201636
E
MALAT1 ASO
IC50 (nM)
n.s.
NAV2729:
YM201636:
F
mCherry
mCherry-VAC14
phase contrast
mCherry
G
mCherry
mCherry + NAV2729
VAC14 OE
VAC14 OE + NAV2729
MALAT1 levels (% control)
[ASO] (nM)
H
MALAT1 ASO
IC50 (nM)
n.s.
VAC14 OE:
NAV2729:

Supplementary Fig. S8: Simultaneous ARF6 and PIKfyve inhibition enhance ASO activity synergistically. (A) *MALAT1* levels in HeLa cells treated with the indicated concentrations of 3-10-3 cEt gapmer targeting *MALAT1* ± SH-BC-893 (5 μM), NAV2729 (12.5 μM), SecinH3 (30 μM), YM201636 (800 nM), or apilimod (100 nM) for 24 h. Mean ± SD shown, n=3-5. **(B)** IC50s of each biological replicate in (A); mean ± SD shown. Due to unequal SD, a Brown-Forsythe and Welch ANOVA test was used with Dunnett's T3 test to correct for multiple comparisons; ***, $p<0.001$; **, $p<0.01$; *, $p<0.05$. **(C)** Viability measured by vital dye (DAPI) exclusion through flow cytometry analysis in HeLa cells treated as in (A). Using an ordinary one-way ANOVA with Tukey's correction for multiple comparisons. Mean ± SD shown, n=3. **(D-E)** Same as (A-B), except in MEFs and NAV2729 and YM201636 in combination. Mean ± SD shown, n=3. **(F)** Phase contrast microscopy or mCherry fluorescence of HeLa cells transduced with pQCXIP-mCherry or mQCXIP-mCherry-VAC14. Scale bar = 20 μm. **(G)** *MALAT1* levels in HeLa cells expressing mCherry or mCherry-VAC14 treated with the indicated concentrations of 3-10-3 cEt gapmer targeting *MALAT1* ± NAV2729 (12.5 μM) for 24 h. Mean ± SD shown, n=3. **(H)** IC50s from each biological replicate in (G); mean ± SD shown. Due to unequal SD, a Brown-Forsythe and Welch ANOVA test was used with Dunnett's T3 test to correct for multiple comparisons; ***, $p<0.001$; **, $p<0.01$. n.s. = not significant.

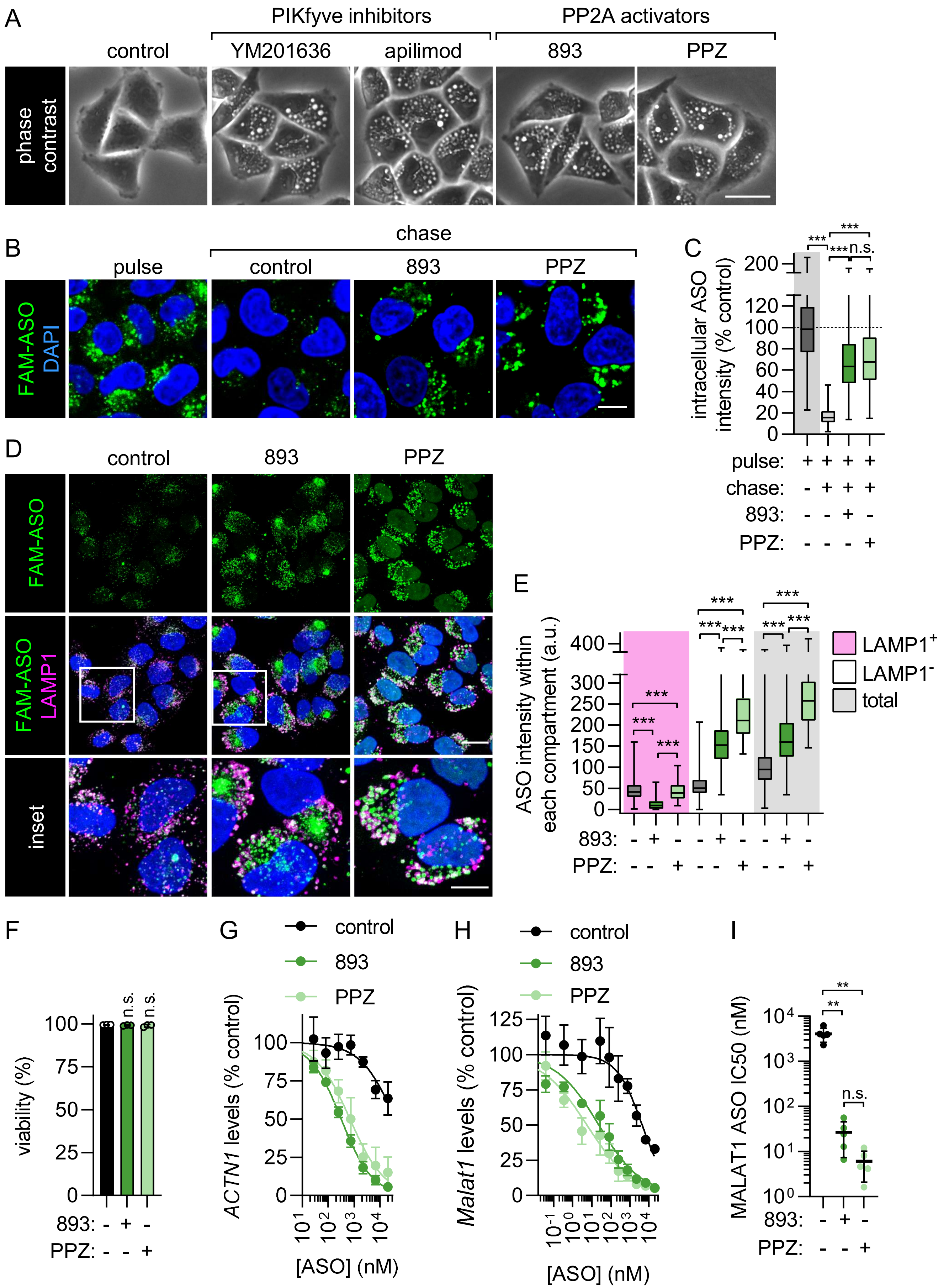
A
PIKfyve inhibitors
PP2A activators
control
YM201636
apilimod
893
PPZ
phase contrast
B
chase
pulse
control
893
PPZ
FAM-ASO DAPI
C
intracellular ASO intensity (% control)
pulse: + + + +
chase: - + + +
893: - - + -
PPZ: - - - +
n.s.
D
control
893
PPZ
FAM-ASO
FAM-ASO LAMP1
inset
E
ASO intensity within each compartment (a.u.)
LAMP1+
LAMP1-
total
893: - + - - + - - + -
PPZ: - - + - - + - - +
F
viability (%)
n.s.
893: - + -
PPZ: - - +
G
control
893
PPZ
ACTN1 levels (% control)
[ASO] (nM)
H
control
893
PPZ
Malat1 levels (% control)
[ASO] (nM)
I
MALAT1 ASO IC50 (nM)
n.s.
893: - + -
PPZ: - - +

Supplementary Fig. S9: PP2A activation blocks endocytic recycling and lysosomal fusion and increases ASO activity. (A) Phase contrast images of HeLa cells treated with YM201636 (800 nM), apilimod (100 nM), SH-BC-893 (5 µM), or PPZ (15 µM) for 3 h. Scale bar = 20 µm. **(B)** HeLa cells were pulsed with FAM-tagged 3-10-3 cEt ASO (2 µM) for 1 h, washed, and then chased in media containing vehicle (DMSO), SH-BC-893 (5 µM), or PPZ (15 µM) for 2 h prior to imaging. **(C)** Quantification of the intracellular ASO fluorescence of cells in (B). At least 100 cells were quantified from each of 2 independent experiments. Because data is not normally distributed, a Kruskal-Wallis ANOVA was used with Dunn's test to correct for multiple comparisons. ***, $p<0.001$. **(D)** FAM-tagged cEt 3-10-3 ASO and LAMP1 localization in HeLa cells treated with SH-BC-893 (5 µM) or PPZ (15 µM) for 6 h. Scale bar = 20 µm. For inset, scale bar = 10 µm. **(E)** Quantification of the raw intensity values for ASO from images in (D) within LAMP2-positive, LAMP2-negative, and total cellular areas. At least 50 cells were quantified from 2 independent experiments. Because data is not normally distributed, a Kruskal-Wallis ANOVA was used with Dunn's test to correct for multiple comparisons. ***, $p <0.001$. **(F)** Viability measured by vital dye (DAPI) exclusion through flow cytometry analysis in HeLa cells treated with SH-BC-893 (5 µM) or PPZ (15 µM) for 24 h. Using an ordinary one-way ANOVA with Tukey's correction for multiple comparisons. Mean ± SD shown, n=3. **(G)** *ACTN1* levels in HeLa cells treated with the indicated concentrations of a 3-10-3 cEt gapmer targeting *ACTN1* ± SH-BC-893 (5 µM) or PPZ (15 µM) for 24 h. Mean ± SD shown, n=3. IC50 for control could not be calculated due to low activity. **(H)** Same as (G), except with 3-10-3 cEt gapmer targeting *Malat1* in MEFs, n=4. **(I)** IC50s from each biological replicate in (H); mean ± SD shown. Using a Welch's t test to correct for unequal SD, **, $p<0.01$. n.s. = not significant.

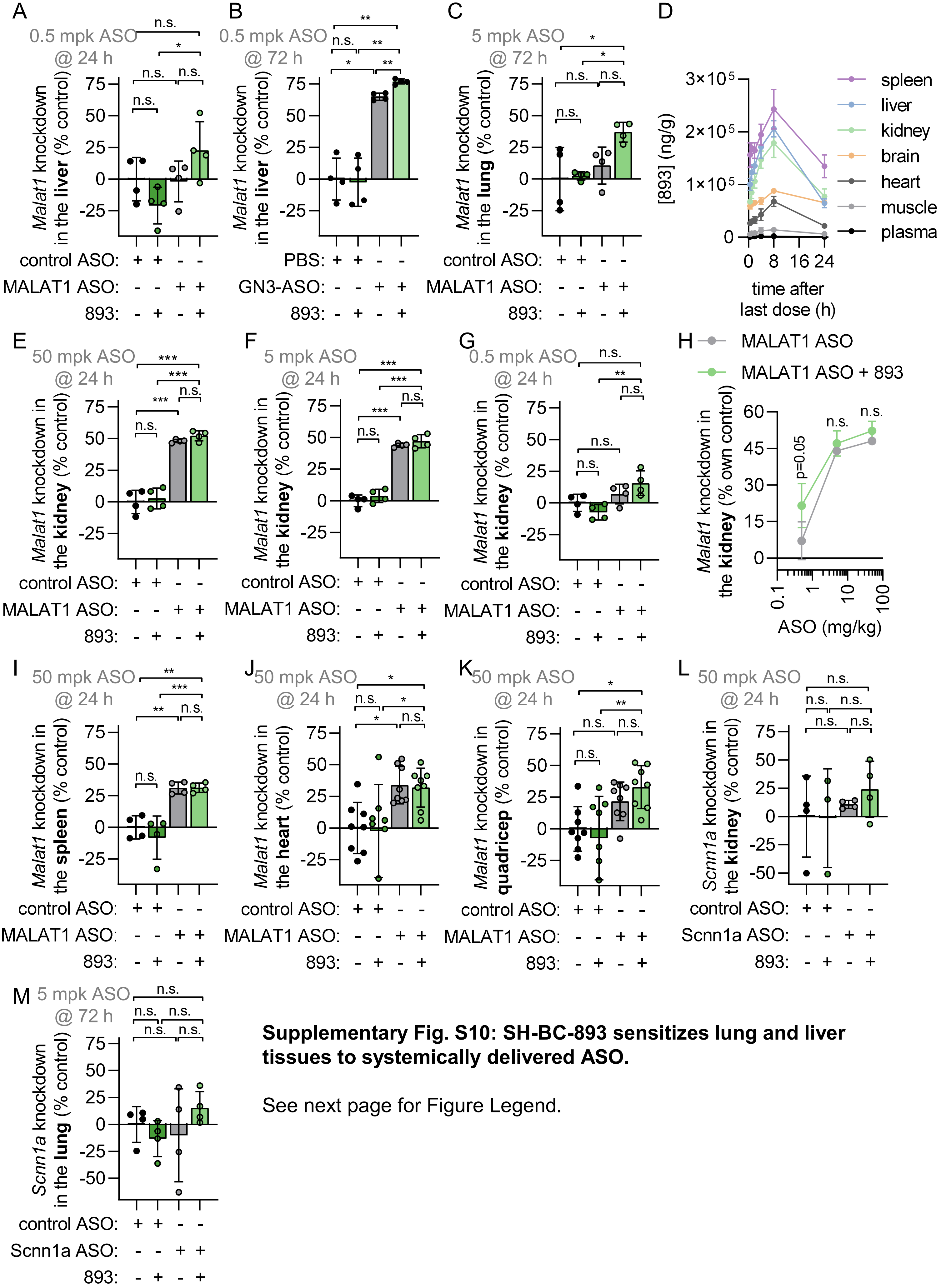

Supplementary Fig. S10: SH-BC-893 sensitizes lung and liver tissues to systemically delivered ASO.

See next page for Figure Legend.

Supplementary Fig. S10: SH-BC-893 sensitizes lung and liver tissues to systemically-delivered ASO. (A) *Malat1* knockdown in the livers of male Balbc/J mice (n=4/group) treated with SH-BC-893 (120 mg/kg P.O.) 2 h before ASO (0.5 mg/kg S.C.) and sacrificed 24 h after a single dose. Non-targeting (control) or *Malat1*-targeting cEt gapmer were used. Mean ± SD shown. Using an ordinary one-way ANOVA with Tukey's correction for multiple comparisons, *, $p<0.05$. **(B)** As in (A), except using a $GalNAc_3$-conjugated (GN) form of the MALAT1 ASO and mice sacrificed 72 h after single dose. Due to unequal SD, a Brown-Forsythe and Welch ANOVA test was used with Dunnett's T3 test to correct for multiple comparisons, *, $p<0.05$; **, $p<0.01$. **(C)** As in (A), except 5 mg/kg ASO and in the lung. **(D)** Tissue SH-BC-893 levels in male (n=3) or female (n=3) CD1 mice treated with 120 mg/kg P.O. Q.D. for 5 d and sacrificed at the indicated time points after the last dose. Mean ± SD shown, n=6. **(E)** As in (A), except 50 mg/kg ASO and kidney tissue evaluated. **(F-G)** As in (E), except 5 mg/kg (F) or 0.5 mg/kg (G) ASO. **(H)** As in (E-G), except expressed as a function of the dose of ASO delivered in mg/kg. Mean ± SD shown (n=4 mice/group). Using an unpaired t-test to compare lung *Malat1* levels in ASO alone or ASO+893 groups, n.s., $p>0.05$. **(I-K)** As in (E), except spleen (I), heart (J), or quadricep muscle (K). **(L)** *Scnn1a* (aka ENaCα) knockdown in the kidney of male Balbc/J mice (n=4 mice/group) treated with SH-BC-893 (120 mg/kg P.O.) 2 h before ASO (50 mg/kg S.C.) and sacrificed 72 h after a single dose. A non-targeting (control) or Scnn1a-targeting cEt gapmer were used. Using an ordinary one-way ANOVA with Tukey's correction for multiple comparisons, ***, $p<0.001$. **(M)** Same as (L), except 5 mg/kg and in lung tissue. n.s. = not significant. RNA levels are expressed relative to the housekeeping gene *Ppia* using the $2^{-\Delta\Delta Ct}$ method. In (A,B,C,E-G,I-M), knockdown is calculated relative to the mean from the mice receiving the non-targeting ASO and water vehicle. In (H), knockdown is expressed relative to the mean from the non-targeting ASO group for either the vehicle- or SH-BC-893-treated mice.

A see *J Clin Invest* (2016) 126:4088. DOI: 10.1172/JCI87148 for detailed analysis of toxicity in mice dosed daily with 120 mg/kg SH-BC-893 for 11 weeks, includes body weight, complete blood chemistry panel, complete blood count, and histology of intestinal crypts.

B see EMBO Mol Med (2021)13:e13086 DOI: 10.15252/emmm.202013086 for a holistic measurement of SH-BC-893 toxicity by monitoring the time spent exercising and the distance ran of mice treated with 120 mg/kg SH-BC-893 P.O. every other day for 4 weeks.

C

Group ID	AST (U/L)	ALT (U/L)	ALP (U/L)	total bilirubin (mg/dL)	conjugated bilirubin (mg/dL)	albumin (g/dL)	globulin (g/dL)	ALB:GLOB ratio	total protein (g/dL)
water PO	85 ± 16	35 ± 8	142 ± 28	0.1 ± 0	0 ± 0	2.4 ± 0.1	1.8 ± 0.1	1.4 ± 0.1	4.1 ± 0.1
120 mg/kg 893 PO	92 ± 44	33 ± 7	138 ± 14	0.2 ± 0.1	0 ± 0	2.4 ± 0.1	1.8 ± 0.1	1.4 ± 0.1	4.2 ± 0.2
240 mg/kg 893 PO	74 ± 17	27 ± 3	125 ± 8	0.1 ± 0.1	0 ± 0	2.4 ± 0.1	1.7 ± 0.1	1.4 ± 0.1	4.1 ± 0.1

Group ID	BUN (mg/dL)	creatinine (mg/dL)	BUN:creatinine ratio	creatine kinase (U/L)	cholesterol (mg/dL)
water PO	21 ± 2	0.1 ± 0.1	171 ± 54	2531 ± 1005	98 ± 5
120 mg/kg 893 PO	25 ± 3	0.1 ± 0	247 ± 34	3914 ± 3298	103 ± 11
240 mg/kg 893 PO	19 ± 2	0.1 ± 0.1	160 ± 45	2243 ± 545	109 ± 14

Group ID	glucose (mg/dL)	calcium (mg/dL)	phosphorus (mg/dL)	bicarbonate (mmol/L)	chloride (mmol/L)	potassium (mmol/L)	sodium (mmol/L)	NA/K Ratio
water PO	310 ± 71	8.4 ± 0.6	9.7 ± 2.3	20 ± 5	110 ± 1	5.8 ± 1.6	146 ± 0	27 ± 5
120 mg/kg 893 PO	314 ± 44	8.3 ± 0.2	9.9 ± 1.0	20 ± 2	109 ± 1	5.5 ± 0.7	144 ± 1	27 ± 4
240 mg/kg 893 PO	375 ± 77	8.3 ± 0.6	10.7 ± 2.4	17 ± 1	109 ± 2	6.2 ± 1.7	144 ± 2	25 ± 6

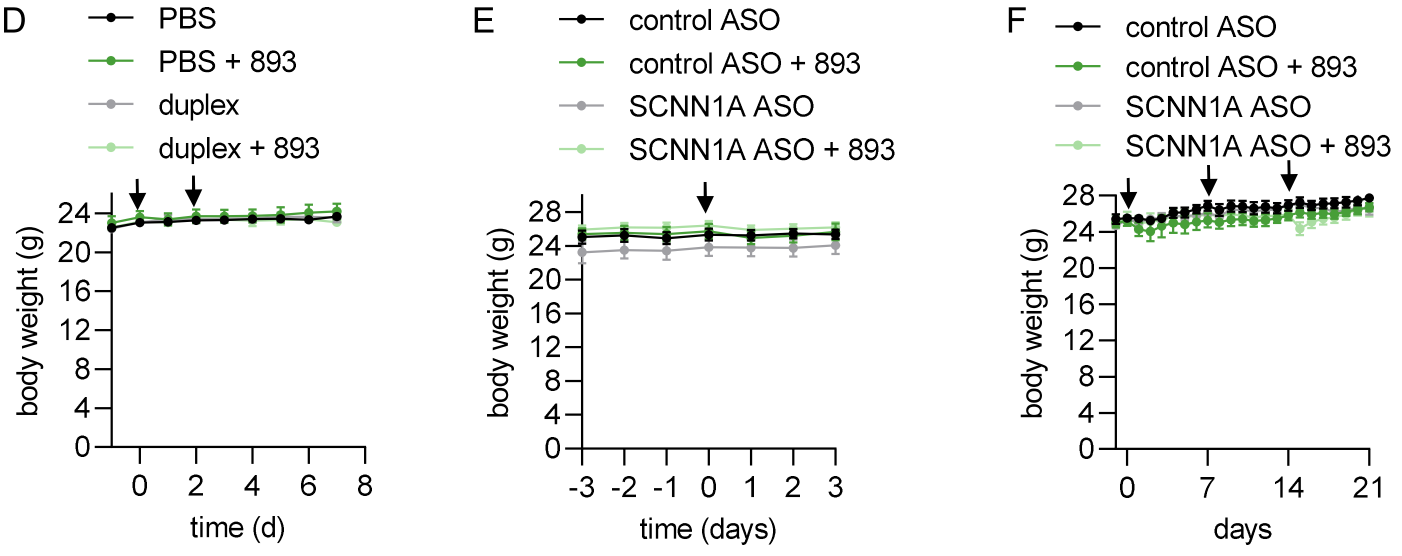

Supplementary Fig. S11: Acute toxicity profile of SH-BC-893 in mice. (A) Reference to publication showing toxicity profile of SH-BC-893 in mice treated with SH-BC-893 (120 mg/kg PO) for 11 weeks. **(B)** Reference to publication showing voluntary exercise in mice treated with SH-BC-893 (120 mg/kg PO) every Monday, Wednesday, and Friday for 4 weeks. **(C)** Blood chemistry in nine-week-old male Balbc/J mice treated with SH-BC-893 (120 or 240 mg/kg PO) and sacrificed 24 h after a single dose to look at acute toxicity. Blood was collected by decapitation and serum separated from whole blood after letting clot in a tube with clot activator gel. Mean ± SD shown, n=4-6 mice/group. **(D-F)** Mouse body weights from experiments in Fig. 5 (D), in Fig. 6E (E), and in Fig. 6G (F). Arrows indicate treatment days.
